# Molecular epidemiology of enterically colonizing *Escherichia coli* with resistance against third-generation cephalosporins isolated from stool samples of European soldiers with concomitant diarrhea on deployment in Western African Mali

**DOI:** 10.3389/fmicb.2023.1169829

**Published:** 2023-05-05

**Authors:** Katharina Hoffmann, Matthias Riediger, Aljoscha Tersteegen, Pauline Marquardt, Sascha Kahlfuß, Achim J. Kaasch, Ralf Matthias Hagen, Hagen Frickmann, Andreas E. Zautner

**Affiliations:** ^1^Institute of Medical Microbiology and Hospital Hygiene, Medical Faculty, Otto-von-Guericke University Magdeburg, Magdeburg, Germany; ^2^Institute of Molecular and Clinical Immunology, Medical Faculty, Otto-von-Guericke University Magdeburg, Magdeburg, Germany; ^3^Health Campus Immunology, Infectiology, and Inflammation (GCI3), Medical Center, Otto-von-Guericke University Magdeburg, Magdeburg, Germany; ^4^CHaMP, Center for Health and Medical Prevention, Otto-von-Guericke-University Magdeburg, Magdeburg, Germany; ^5^Department of Microbiology and Hospital Hygiene, Bundeswehr Central Hospital Koblenz, Koblenz, Germany; ^6^Department of Microbiology and Hospital Hygiene, Bundeswehr Hospital Hamburg, Hamburg, Germany; ^7^Institute for Medical Microbiology, Virology and Hospital Hygiene, University Medicine Rostock, Rostock, Germany

**Keywords:** extended spectrum beta lactamase, ESBL, Enterobacterales, antibiotic resistance, surveillance, Sub-Saharan Africa, Mali, military personnel

## Abstract

Extended spectrum beta-lactamases (ESBL) are frequently found in Enterobacterales isolates from Western Africa. However, information on the molecular epidemiology of regional ESBL-positive Enterobacterales strains is scarce. In order to provide epidemiological information, ESBL-positive *Escherichia coli* isolates from stool samples of European soldiers with diarrhea deployed to a field camp in Mali were subjected to whole-genome sequencing (Illumina MiSeq and Oxford Nanopore MinION) and antimicrobial susceptibility testing. With two exemptions, sequence-based analysis suggested an absence of transmission events between soldiers as indicated by a high genetic diversity of isolates and sequence types, confirming previous rep-PCR results. Third-generation cephalosporin resistance was associated with the presence of *bla*_CTX-M-15_ genes with (*n* = 14) and without (*n* = 5) co-occurring *bla*_TEM-1b_ genes. Between 0 and 6 virulence and resistance plasmids per isolate were recorded. The detected resistance plasmids could be categorized into five types, which, in turn, share different sequence-identical segments, representing particular antimicrobial resistance gene-associated mobile genetic elements (MGEs). Phenotypic resistance rates within the 19 assessed isolates that showed distinguishable colony morphologies were 94.7% (18/19) against ampicillin-sulbactam and trimethoprim/sulfamethoxazole, 68.4% (13/19) against moxifloxacin, 31.6% (6/19) against ciprofloxacin, 42.1% (8/19) against gentamicin, 31.6% (6/19) against tobramycin, and 21.1% (4/19) against piperacillin-tazobactam and fosfomycin. Virulence-associated genes mediating infectious gastroenteritis were rarely detected. The gene *aggR*, which is characteristic for enteroaggregative *E. coli*, was only detected in one single isolate. In summary, we found a variety of different strains and clonal lineages of ESBL-carrying *E. coli.* Transmission either between soldiers or from common contaminated sources was demonstrated in two cases and played only a minor role in this military field camp, while there were indications that resistance gene bearing MGEs had been exchanged between antimicrobial resistance gene-(ARG-)carrying plasmids.

## 1. Introduction

Antimicrobial resistance (AMR) is quite common in Sub-Saharan Africa, particularly in Gram-negative rods, such as Enterobacterales (Tompkins et al., 2021). To counteract this challenge, the implementation of antibiotic stewardship programs in Africa has been suggested (Akpan et al., 2020). However, evidence-guided optimized prescription of antimicrobial therapy requires knowledge on regionally common susceptibility patterns. Therefore, AMR surveillance is crucial for this purpose (Akpan et al., 2020).

Antimicrobial resistance surveillance data from war- and crisis-haunted Sub-Saharan regions like Western African Mali are largely unavailable. Data on antimicrobial resistance and susceptibility mainly arise from arbitrarily distributed, small prevalence assessments. Next to information on extended spectrum beta-lactamase- (ESBL-)positive Enterobacterales (Sangare et al., 2015), resistance rates for other microorganisms such as *Neisseria gonorrhoeae* (Jary et al., 2021), pneumococci (Campbell et al., 2004), *Haemophilus influenzae* (Pécoul et al., 1991; Sow et al., 2005), and *Staphylococcus aureus* (Ruimy et al., 2008) are also scarce. In comparison to the latter, Malian studies on Enterobacterales with resistance against third-generation cephalosporins are generally more frequent (Sangare et al., 2015; Nkansa-Gyamfi et al., 2019). In a Malian orphanage, ESBL-positive Enterobacterales colonized the inhabitants’ and the workers’ skin to a proportion that they could be virtually considered as standard bacterial flora (Tandé et al., 2009). Consequently, in case of international adoption, nosocomial spread of such bacteria is a frequently observed phenomenon (Tandé et al., 2010). While such colonization was usually harmless in immunocompetent individuals (Tandé et al., 2009), however, immunocompromised hosts with tuberculosis and acquired immunosuppression suffered from severe superinfection due to ESBL-positive *Klebsiella* spp. (Meli et al., 2020). Among Enterobacterales isolated from Malian blood cultures, ESBL-carriage in about two out of three isolates has been repeatedly described (Sangare et al., 2016, 2017; Sangaré et al., 2017). Next to typical ESBL-harboring Enterobacterales like *Escherichia* spp. and *Klebsiella* spp., ESBL enzymes are also common in *Salmonella enterica* in Mali and in the Senegal (Boisramé-Gastrin et al., 2011; Harrois et al., 2014).

In a surveillance study conducted between December 2013 and August 2014 with European soldiers deployed to Mali on the European Union Training Mission (EUTM), ESBL-positive Enterobacterales were isolated from 13 out of 48 stool samples obtained from patients with diarrhea during the study interval (Frickmann et al., 2015). With the exemption of a single *Klebsiella pneumoniae* isolate, only ESBL-positive *E. coli* isolates were detected indicating multiple colonization events in superficial rep-PCR-based typing (Frickmann et al., 2015). The multiple transmission events were likely to be linked to environmental sources, since ESBL-positive Enterobacterales were detected in environmental samples and also in the food sources of the mission headquarters (Maaßen et al., 2017; Frickmann et al., 2018a). Genes from the *bla*_CTX-M_ group 1 were reported to be responsible for the observed third-generation cephalosporin resistance (Hagen et al., 2015). Despite high enteric colonization rates of the European soldiers on deployment in Mali with ESBL-positive Enterobacterales (Frickmann et al., 2015), low detectable colonization of about 7% (3/43 samples) was recorded in German military returnees during screenings in the course of returnee assessments about 3 months after deployment to Mali (Frickmann et al., 2016; Pankok et al., 2022a). This finding is well in line with previous reports indicating that enteric colonization with ESBL-positive Enterobacterales is transient and that rates of detection considerably drop in the first 3 months after returning from international journeys (Ruppé et al., 2015; Arcilla et al., 2017; Frickmann et al., 2018b).

In our study, we performed whole-genome sequencing of ESBL-positive *E. coli* isolates cultured from the stool samples of the European soldiers deployed to Mali (Frickmann et al., 2015; Hagen et al., 2015) and analyzed the epidemiological relationship between isolates and the presence of molecular resistance and virulence determinants. In addition to virulence factors and antimicrobial resistance genes (ARGs) that are chromosomally encoded, the focus was on the analysis of antimicrobial resistance gene-(ARG-) bearing resistance plasmids. The potential abundance of highly virulent clonal lineages of *E. coli* was addressed (Denamur et al., 2021). Accordingly, our study provides important additional information on the local epidemiology in Mali.

## 2. Materials and methods

### 2.1. Study site and origin of the isolates

**Figure 1:**
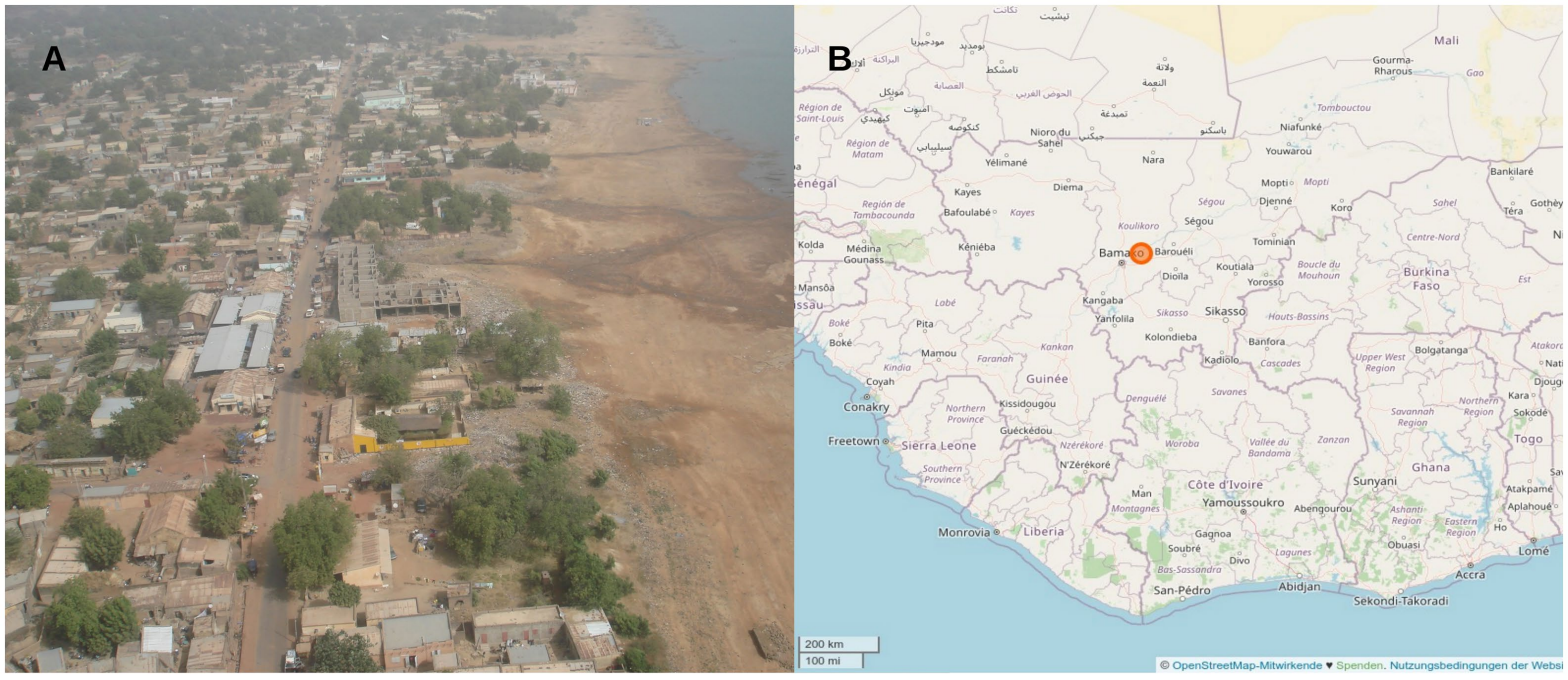
**(A)** Aerial view of Koulikoro, where the main training site of the EUTM mission was located. **(B)** Location of the study site ca. 60 km north-east from the Malian capital Bamako.

*Escherichia coli* isolates with resistance against third-generation cephalosporins had been collected in a previous surveillance study, which assessed stool samples of European soldiers with diarrhea during their deployment in Mali between December 2013 and August 2014, by isolation from Brilliance ESBL selective agar (Oxoid, Basingstoke, United Kingdom) as described before (Frickmann et al., 2015). Brilliance ESBL selective agar does not specifically select diarrheagenic *E. coli* and culture-based screening for them was not the purpose of the study approach. Forty-eight diarrhea stool samples were collected in the military Camp of Koulikoro about 60 km from the Malian capital Bamako, where the training site of the EUTM Mali mission was located. Koulikoro is a small community at the river Niger with about 50,000 inhabitants (latitude and longitude 12.52 N 7.34 W, Figure 1). During the sampling period, about 600 European soldiers were deployed on the EUTM Mali mission and changed about every 4–6 months.

The strains had been isolated at the Department of Tropical Medicine at the Bernhard Nocht Institute, Bundeswehr Hospital Hamburg, Germany and stored as Microbank™-cryostocks (Pro-Lab Diagnostics, Round Rock, Texas, United States) at −80°C in the strain collection of the Department of Microbiology and Hospital Hygiene, Bundeswehr Central Hospital Koblenz, Germany, which is in charge for population-based microbiological assessments and deployment-associated microbiology in the German armed forces. Superficial molecular assessment of the isolates by rep-PCR typing had been initially performed after sampling (Frickmann et al., 2015; Hagen et al., 2015). Cultivation prior to DNA extraction was performed on Columbia agar enriched with 5% sheep blood at 37°C in a CO_2_-enriched atmosphere for 24–48 h.

### 2.2. Mass-spectrometry based species identification and phenotypic resistance testing

At the Medical Faculty of the Otto-von-Guericke University Magdeburg, all isolates were confirmed as *E. coli* by matrix-assisted laser-desorption-ionization time-of-flight mass-spectrometry (MALDI-TOF-MS) using a Vitek MS mass spectrometer with the corresponding VITEK MS Knowledge Base V3.2 (bioMérieux, Mary-l’Étoile, France). Phenotypic susceptibility testing was performed using the *Micronaut* Microdilution System (MERLIN Diagnostika GmbH, Bornheim-Hersel, Germany) employing MCN-S MH Hannover GN3 plates (E1-247-100) according to the manufacturer’s instructions. The results were interpreted according to the European Committee on Antimicrobial Susceptibility Testing (EUCAST) V12.0 (2022) standard.

### 2.3. DNA extraction, library preparation, and whole-genome sequencing

Isolation of bacterial DNA was performed *via* the cetyltrimethylammonium bromide (CTAB) method as described previously by Bechmann et al. (2022). Library preparation for Illumina paired-end sequencing was performed using the TruePrep DNA Library Prep Kit V2 for Illumina (1°ng; Vazyme Biotech Co. Ltd., Nanjing, China). Libraries were barcoded using the Nextera XT Index Kit (Illumina, San Diego, United States) and sequenced using the MiSeq Reagent Kit v2 (500-cycles, Illumina) as described by the manufacturer. Barcoded libraries for Nanopore long-read sequencing were prepared using the Rapid Barcoding Kit 96 (SQK-RBK110.96) according to the manufacturer’s instructions and sequenced on a R9.4.1 flow cell (FLO-MIN106) on the MinION platform (Oxford Nanopore technologies ltd., Oxford, United Kingdom).

### 2.4. Genome assembly

Illumina paired-end reads were preprocessed using *fastp* (https://github.com/OpenGene/fastp, v0.23.2), and *filtlong* (*parameters: --min_length 1,000 --keep_percent 95*, https://github.com/rrwick/Filtlong, v0.2.1) was used for long reads. Genomes were assembled using a long read consensus assembly approach presented in the *trycycler software package* (https://github.com/rrwick/Trycycler, v0.5.3), and cross-validated with results from the short read assembler *SKESA* (Shovill/SKESA, v2.4.0; Souvorov et al., 2018), as well as the hybrid assembler *unicycler* (https://github.com/rrwick/Unicycler, v0.4.8). In short, long reads were sampled into 20 unique subsets of reads with a 100x sequencing depth and each sets of four subsets were independently assembled using canu v1.8.1 (Koren et al., 2017), *flye v2.9.1-b1780* (Kolmogorov et al., 2019), *miniasm v0.3*,^1^ *minipolish v0.1.3* (Wick and Holt, 2019), *necat* v0.0.1,^2^ and *raven* v1.8.1^3^ to generate 20 independent long-read assemblies. Subsequently, the *trycycler* functions *cluster*, *reconcile*, *msa*, *partition*, and *consensus* were applied on the set of assemblies for each sample to generated consensus assemblies. Finally, the consensus assemblies were polished using the long read polisher *medaka*^4^ and short read polisher *polypolish* v0.5.0^5^ (Wick and Holt, 2022).

### 2.5. Annotations

The final assemblies were annotated using the NCBI Prokaryotic Genome Annotation Pipeline (Tatusova et al., 2016; Haft et al., 2018; Li et al., 2021) and further characterizations comprised plasmid identification and annotation (MOB-suite, PlasmidFinder, MobileElementFinder; Camacho et al., 2009; Carattoli et al., 2014; Robertson and Nash, 2018; Johansson et al., 2021), antimicrobial resistance (AMR) gene detection (AMRFinderPlus v3.11.2; Resfinder v4.1, PointFinder, ResFinder FG V1.0; Sommer et al., 2009; Zankari et al., 2012, 2017; Pehrsson et al., 2016; Feldgarden et al., 2021), and virulence gene detection (Virulence Finder; Joensen et al., 2014; Malberg Tetzschner et al., 2020).

### 2.6. Core genome multi locus sequence typing

Core genome multi-locus sequence typing (cgMLST) from assembled *E. coli* genomes was conducted using 2,520 cgMLST tragets provided by Ridom SeqSphere+ (v8.5.1,_2022–11). (I) MST for 16 samples was based on 2,520 columns, pairwise ignoring missing values (Jünemann et al., 2013; Weber et al., 2019) with a logarithmic scale. Distance was based on columns from *E. coli* cgMLST (2513) and *E.coli* MLST by Warwick (7). (II) MST for 55 samples (including reference datasets from Niger, Ghana, and Germany) was based on 2,274 columns, pairwise ignoring missing values. Distance was based on columns from *E. coli* cgMLST (2,274).

### 2.7. Ethics statement

Ethical clearance for the assessment of the surveillance data in an anonymized way without requirement of informed consent was provided by the ethics committee of the medical association of Hamburg (WF-037/14) on 20th January 2015 in line with National German laws. The study was conducted according to the guidelines of the Declaration of Helsinki.

## 3. Results

### 3.1. Phenotypic resistance patterns

From 12 of 48 soldiers (25%) with diarrhea on the EUTM mission, a total of 19 *E. coli* isolates were selected from ESBL screening agar based on the exhibited specific colony morphology. Thereby, the number of different morphotypes ranged from 1 to 4, comprising eight individuals with one morphotype, two individuals with two morphotypes, and one individuum each with three and with four morphotypes, respectively. All isolates were confirmed as *E. coli* by matrix-assisted laser-desorption-ionization time-of-flight mass-spectrometry (MALDI-TOF-MS). Automated antimicrobial susceptibility testing using VITEK-2 AST-N263 cards with interpretation according to the EUCAST 2022 standard confirmed ESBL phenotypes in all instances (positive ESBL screening test). Resistance in 100% (19/19) of the isolates was recorded against ampicillin, cefuroxime, cefotaxime, and ceftazidime; 94.7% (18/19) against ampicillin-sulbactam and trimethoprim/sulfamethoxazole (5.3% = 1/19 susceptible a high dosage), 68.4% (13/19) against moxifloxacin, 42.1% (8/19) against gentamicin, 31.6% (6/19) against ciprofloxacin (26.3% = 5/19 susceptible at high dosage) and tobramycin, and 21.1% (4/19) against piperacillin-tazobactam and fosfomycin. Thus, a high percentage of piperacillin-tazobactam susceptible and trimethoprim/sulfamethoxazole resistant ESBL-isolates was observed. All isolates (19/19) were susceptible toward imipenem, meropenem, ertapenem, tigecycline, and colistin. Details on the susceptibility patterns of the isolates are provided in Table 1.

**Table 1:** Phenotypic resistance as measured for the assessed *Escherichia coli* isolates applying MICRONAUT microdilution broth plates (catalog number: E1-247-100) interpreted according to the EUCAST 2022 standard.

Isolate	ESBL (+/−)	AMP (MIC)	SAM (MIC)	TZP (MIC)	CXM (MIC)	CTX (MIC)	CAZ (MIC)	IMP (MIC)	MER (MIC)	ERT (MIC)	CIP (MIC)	MOX (MIC)	GEN (MIC)	TOB (MIC)	SXT (MIC)	FOS (MIC)	TGC (MIC)	COL (MIC)
MLI023_1	+	R (>32)	R (>16/4)	S (2/4)	R (>16)	R (>8)	R (32)	S (≤1)	S (≤0.125)	S (≤0.125)	S (0.25)	R (>0.5)	S (1)	S (≤0.5)	R (>8/152)	R (128)	S (≤0.25)	S (≤0.5)
MLI023_2	+	R (>32)	R (>16/4)	S (4/4)	R (>16)	R (>8)	R (>32)	S (≤1)	S (≤0.125)	S (≤0.125)	I (0.5)	R (>0.5)	S (1)	S (≤0.5)	R (>8/152)	S (≤16)	S (≤0.25)	S (≤0.5)
MLI102	+	R (>32)	R (>16/4)	S (2/4)	R (>16)	R (>8)	R (16)	S (≤1)	S (≤0.125)	S (≤0.125)	S (≤0.0625)	S (0.125)	S (≤0.5)	S (≤0.5)	R (>8/152)	R (128)	S (≤0.25)	S (≤0.5)
MLI104_1	+	R (>32)	R (>16/4)	S (2/4)	R (>16)	R (>8)	R (16)	S (≤1)	S (≤0.125)	S (≤0.125)	I (0.5)	R (>0.5)	S (1)	S (≤0.5)	R (>8/152)	S (≤16)	S (≤0.25)	S (≤0.5)
MLI104_2	+	R (>32)	R (>16/4)	S (1/4)	R (>16)	R (>8)	R (8)	S (≤1)	S (≤0.125)	S (≤0.125)	S (≤0.0625)	S (≤0.0625)	R (>8)	S (2)	R (>8/152)	R (64)	S (≤0.25)	S (≤0.5)
MLI105	+	R (>32)	R (16/4)	S (4/4)	R (>16)	R (>8)	R (32)	S (≤1)	S (≤0.125)	S (≤0.125)	S (0.125)	S (0.25)	S (1)	S (1)	R (>8/152)	R (64)	S (0.5)	S (≤0.5)
MLI106_1	+	R (>32)	R (16/4)	S (1/4)	R (>16)	R (>8)	R (32)	S (≤1)	S (≤0.125)	S (0.25)	I (0.5)	R (>0.5)	S (1)	S (≤0.5)	R (>8/152)	S (32)	S (≤0.25)	S (≤0.5)
MLI106_2	+	R (>32)	R (>16/4)	R (32/4)	R (>16)	R (>8)	R (>32)	S (≤1)	S (≤0.125)	S (≤0.125)	R (>2)	R (>0.5)	R (>8)	R (>8)	R (>8/152)	S (≤16)	S (≤0.25)	S (≤0.5)
MLI106_3	+	R (>32)	R (16/4)	S (1/4)	R (>16)	R (>8)	R (16)	S (≤1)	S (≤0.125)	S (0.25)	I (0.5)	R (>0.5)	S (≤0.5)	S (≤0.5)	R (>8/152)	S (32)	S (≤0.25)	S (≤0.5)
MLI106_4	+	R (>32)	R (>16/4)	R (32/4)	R (>16)	R (>8)	R (>32)	S (≤1)	S (≤0.125)	S (≤0.125)	R (>2)	R (>0.5)	R (>8)	R (>8)	R (>8/152)	S (≤16)	S (≤0.25)	S (≤0.5)
MLI107	+	R (>32)	S (2/4)	S (1/4)	R (>16)	R (>8)	R (8)	S (≤1)	S (≤0.125)	S (≤0.125)	S (0.25)	S (≤0.0625)	S (≤0.5)	S (1)	I (4/76)	S (≤16)	S (≤0.25)	S (≤0.5)
MLI108_1	+	R (>32)	R (>16/4)	R (16/4)	R (>16)	R (>8)	R (>32)	S (≤1)	S (≤0.125)	S (≤0.125)	R (>2)	R (>0.5)	R (>8)	R (>8)	R (>8/152)	S (≤16)	S (≤0.25)	S (≤0.5)
MLI108_2	+	R (>32)	R (>16/4)	R (16/4)	R (>16)	R (>8)	R (32)	S (≤1)	S (≤0.125)	S (≤0.125)	R (>2)	R (>0.5)	R (>8)	R (>8)	R (>8/152)	S (≤16)	S (≤0.25)	S (≤0.5)
MLI108_3	+	R (>32)	R (>16/4)	S (4/4)	R (>16)	R (>8)	R (>32)	S (≤1)	S (≤0.125)	S (≤0.125)	R (>2)	R (>0.5)	R (8)	R (8)	R (>8/152)	S (≤16)	S (≤0.25)	S (≤0.5)
MLI109	+	R (>32)	R (>16/4)	S (4/4)	R (>16)	R (>8)	R (32)	S (≤1)	S (≤0.125)	S (≤0.125)	R (>2)	R (>0.5)	R (>8)	R (>8)	R (>8/152)	S (≤16)	S (≤0.25)	S (≤0.5)
MLI110	+	R (>32)	R (>16/4)	(S) 2/4	R (>16)	R (>8)	R (16)	S (≤1)	S (≤0.125)	S (≤0.125)	I (1)	R (>0.5)	R (8)	S (1)	R (>8/152)	S (≤16)	S (≤0.25)	S (≤0.5)
MLI114	+	R (>32)	R (16/4)	S (1/4)	R (>16)	R (>8)	R (8)	S (≤1)	S (≤0.125)	S (≤0.125)	S (0.125)	R (0.5)	S (1)	S (≤0.5)	R (>8/152)	S (≤16)	S (≤0.25)	S (≤0.5)
MLI121	+	R (>32)	R (>16/4)	S (2/4)	R (>16)	R (>8)	R (16)	S (≤1)	S (≤0.125)	S (≤0.125)	S (0.25)	S (0.25)	S (≤0.5)	S (≤0.5)	R (>8/152)	S (≤16)	S (≤0.25)	S (≤0.5)
MLI124	+	R (>32)	S (2/4)	S (2/4)	R (>16)	R (>8)	R (16)	S (≤1)	S (≤0.125)	S (≤0.125)	S (0.25)	R (0.5)	S (1)	S (≤0.5)	R (>8/152)	S (≤16)	S (≤0.25)	S (≤0.5)

Abbreviations were chosen adapted from https://journals.asm.org/journal/aac/abbreviations (last accessed on 11th March 2022). ESBL, extended spectrum beta-lactamase; MIC, minimum inhibitory concentration; AMP, ampicillin; SAM, ampicillin-sulbactam; CAZ, ceftazidime; CIP, ciprofloxacin; COL, colistin; CTX, cefotaxime; CXM, cefuroxime; ERT, ertapenem; FOS, fosfomycin; GEN, gentamicin; IMP, imipenem; MEM, meropenem; MOX, moxifloxacin; TZP, piperacillin-tazobactam; SXT, trimethoprim-sulfamethoxazole; TGC, tigecycline; TOB, tobramycin; S, susceptible; I, susceptible a high dosage; R, resistant. MIC values in mg/L. n.i., not interpretable. ESBL status was defined based on the results of VITEK-2 AST-N263 cards. Color codes comprise red = resistant, yellow = susceptible at a higher dose of the drug, green = susceptible, and blue = not interpretable. The order of the isolates is defined by anonymized patient codes as already used in a previous study (Frickmann et al., 2015).

### 3.2. cgMLST and clonal lineages of the isolates

**Figure 2:**
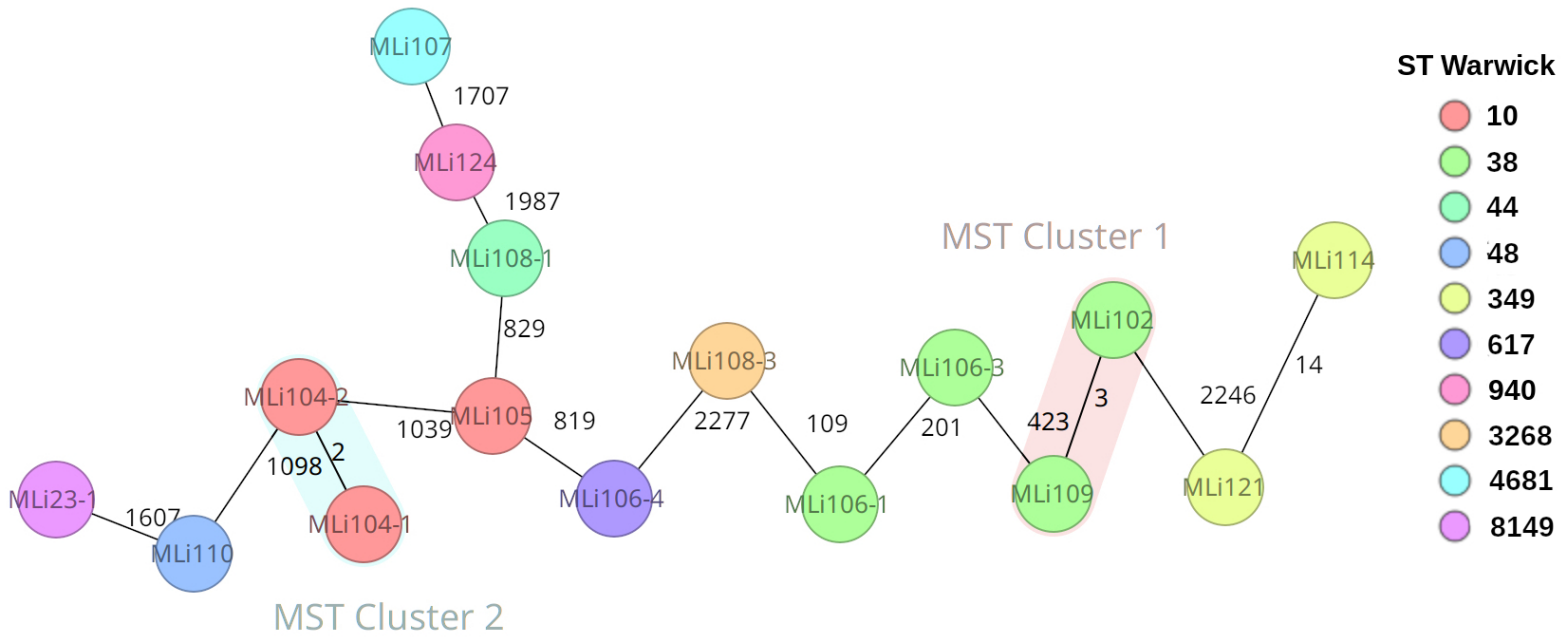
Ridom SeqSphere+ generated cgMLST-based minimum spanning tree showing the genetic distance of the *Escherichia coli* isolates indicated by numbers of allele differences in a total of 2,520 assessed columns (*E. coli* cgMLST: 2513; pairwise ignoring missing values, logarithmic scale, *E. coli* MLST Warwick: 7; cluster distance threshold 10). The nodes are colored by ST Warwick (see legend).

Assemblies of 16 isolates from the 12 ESBL positive soldiers were investigated *via* cgMLST-based analysis to exclude an outbreak. The recorded phylogenetic distance between the isolates ranged from 2 to 2,277 alleles as indicated by the cgMLST-based minimum spanning tree. Three pairs of isolates turned out to have the same cgMLST-based sequence type as well as on closer analysis the exact same genome sequence and were therefore considered identical, i.e., MLI23-1 and -2 (in spite of varying phenotypic susceptibility testing results, Table 1), MLI106-2 and -4, and MLI108-1 and -2 are copy strains; therefore, only one isolate was considered in the phylogenetic analysis (Figure 2). Genetically identical isolates, for which only colony-morphology had been different, were associated with the same soldier in all three instances. The isolates MLI104-1 and -2 originating from a single individual are very closely related, which is indicated by a difference of only two alleles. Close phylogenetic relationship between isolates from two different individuals indicative of direct transmission or common sources was detected in two cases. Thus, the isolates MLI114 and MLI121 differed by only 14 alleles and the isolates MLI102 and MLI109 differed by only three alleles within the 2,520 considered columns of the cgMLST scheme. Of note, the isolates MLI102 and MLI109 had been collected from samples taken within a 10-day-interval. For MLI114 and 121, details on a timely association cannot be provided due to incomplete data acquisition during the epidemiological assessment (Frickmann et al., 2015). The remaining isolates showed high genetic diversity, indicating that they originated from different sources and were not transmitted from soldier to soldier. This high diversity was confirmed by the high number of detected sequence types as shown in the Supplementary Table 1.

In order to gain at least an orienting impression of whether the isolates we analyzed originated in West Africa/Mali or were imported from Europe by the soldiers, we performed a further cgMLST analysis. Since there were no *E. coli* sequence data from Mali in Enterobase and NCBI GenBank so far, and no such sequence data were available from most neighboring countries either, we included a dozen *E. coli* sequences each from Niger and Ghana and 24 *E. coli* sequences from two different studies from Germany in a second cgMLST analysis (Supplementary Table 3; Supplementary Figure 1). In this analysis, only four clusters showed up at a cluster distance threshold of 10. These clusters include only two isolates each. These are the two clusters from our study mentioned above as well as one cluster formed by two isolates from Ghana and one cluster formed by two isolates from Germany. This means there was actually no evidence of importation of the *E. coli* isolates we considered in the study from Europe. Taking a higher perspective and looking at the studied isolates by origin in our cgMLST-based dendrogram, we recognized three superclusters. Thus, we found a supercluster on the right side of the tree of predominantly German isolates in which there were still three isolates from Ghana. The distance to the next isolate from Mali is 2,135 alleles. On the left side of the dendrogram is another cluster of predominantly German isolates together with isolate MLI124. The distance of this supercluster to the next isolate from Africa/Ghana is 1,556 alleles. In between is a supercluster containing 18 of 19 isolates from Mali and, in addition, all isolates/sequence types from Niger and eight of 11 sequence types from Ghana. Only two isolates from the German datasets are in this supercluster belonging to the sequence types ST 10 (17–04317) and ST 216 (17–041045), which are both globally distributed lineages. In summary, according to the limitations of the method, it can be assumed that the majority of the isolates really originated from Mali and only for isolate MLI124 there is a suspicion that it was imported from Europe.

### 3.3. Identified antimicrobial resistance genes

**Figure 3:**
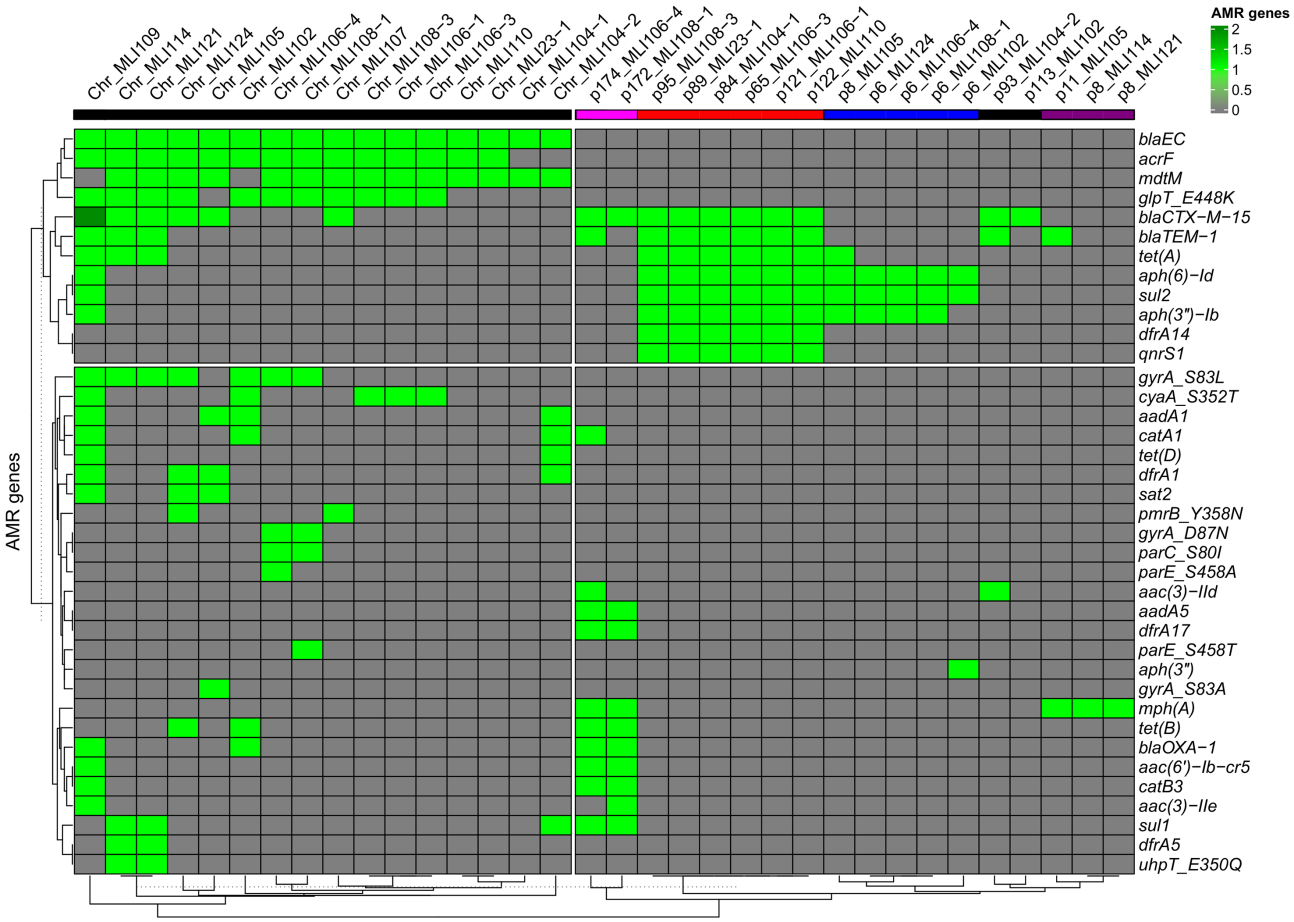
Heatmap of the associations of bacterial chromosomes (left side) as well as plasmids (right side) with encoded ARGs. The heatmap with the ARG/chromosome/plasmid sequence-based dendrograms were generated using the R package ComplexHeatmap [hierarchical clustering (hclust) with Euclidean distance]. A light green rectangle indicates the presence of a specific ARG, a dark green rectangle indicates two copies of a specific ARG. A gray rectangle indicates the absence of a particular ARG. The five different plasmid types, based on gene overlaps among each other, are indicated by different colored bars in the header of the heat map: type 1—pink, type 2—red, type 3—blue, type 4—black, and type 5—purple.

In all 16 isolates (excluding the copy strains), third-generation cephalosporin resistance could be attributed either to *bla*_CTX-M-15_ alone (*n* = 4/16) or in combination with *bla*_TEM-1b_ (*n* = 12/16). Details are provided in Figure 3, Supplementary Figure 2, and Supplementary Table 1, next to information on other identified antimicrobial resistance genes either encoded on the bacterial chromosomes or on plasmids. As shown there, the resistance-mediating genes *bla*_EC_ and *bla*_CTX-M-15_ were the only ones occurring in all isolates. A fourth beta-lactamase gene *bla*_OXA-1_ could be detected in four isolates.

Of the isolates tested, 11/16 were found to be moxifloxacin resistant. Of the quinolone resistance genes detected, the vast majority were found in these 11 moxifloxacin resistant isolates: *qnrS1* (7/11), *gyrA* S83L (7/11), *gyrA* D87N (4/11), *parE* S458T (2/11), *parE* S458A (1/11), and *parC* S80I (2/11). None of the moxifloxacin resistant isolates did not have at least one of these quinolone resistance determinants. Two of the moxifloxacin resistant isolates (MLI114 and MLI 124) were ciprofloxacin susceptible. Both isolates were positive for *gyrA* S83L only. Nevertheless, among the five quinolone susceptible isolates, there was one isolate (MLI105) that was *gyrA* S83A positive and one (MLI121) that was *gyrA* S83L positive.

Resistance to trimethoprim-sulfamethoxazole was attributable to the combined occurrence of the sulfonamide resistance genes *sul1* (*n* = 5) and *sul2* (*n* = 12) as well as the trimethoprim resistance genes *dfrA1* (*n* = 4), *dfrA5* (*n* = 2), *dfrA14* (*n* = 6), and *dfrA17* (*n* = 2). The only isolate that was trimethoprim-sulfamethoxazole susceptible at increased dosage, *E. coli* MLI107, did not exhibit any of these six ARGs.

Of the six gentamicin resistant isolates, five were positive for *aph6-Id* and *aph3-Ib*. In one isolate, *aac3-IId*, *aadA5*, and *aac6-Ib-cr5* were found additionally. In a second isolate, *aac3-IIe*, *aadA5*, and *aac6-Ib-cr5*; and in a third *aac3-IIe* and *aac6-Ib-cr5* were additional aminoglycoside-specific ARGs. In a single gentamicin-resistant isolate, *aac3-IId* was the only aminoglycoside-specific ARG.

Antimicrobial resistance genes conferring tetracycline resistance, which was not phenotypically tested, were *tetA* (*n* = 10), *tetB* (*n* = 4), and *tetD* (*n* = 2).

The *pmrB* gene, associated with polymyxin B resistance, was detected chromosomally encoded in two isolates. However, we only tested susceptibility to colistin (polymyxin E) and did not find phenotypically detectable resistance.

### 3.4. Resistance plasmids

As indicated in Figure 3, in the isolates investigated five different types of resistance plasmids were found according to the ARGs that were encoded on them. These numerical plasmid types assigned by us correlate only partially with the incompatibility types listed for each plasmid in Supplementary Table 1. The plasmids of the first plasmid type had a molecular weight of more than 170 kbp, belonged to the incompatibility types IncFIA, IncFIB, as well as IncFII and encoded the type-common resistance genes *bla*_CTX-M-15_, *aadA5*, *dfrA17*, *mphA*, *tetB*, *bla*_OXA-1_, *aac*(*6′*)*-Ib-cr5*, *catB3*, and *sul1*. The plasmids of the second plasmid type had a molecular weight of 65–122 kbp, belonged to the incompatibility types IncFIB or IncY and encoded the type-common ARGs *bla*_CTX-M-15_, *bla*_TEM-1_, *tetA*, *aph*(*6*)*-Id*, *sul2*, *aph*(*3″*)*-*(*Ib*), *dfrA14*, and *qnrS1*. The plasmids of the third plasmid type were much smaller, belonged to none of the incompatibility types, and had a molecular weight of 6–8 kbp and encoded the type-common ARGs *aph*(*6*)*-Id*, *sul2*, and *aph*(*3″*)*-*(*Ib*). The plasmids of the fourth plasmid type belonged to the incompatibility type IncI1 or to none incompatibility type, and had *bla*_CTX-M-15_ as the only common ARG at a molecular weight of 93–113 kbp, and the plasmids of the fifth plasmid type were again much smaller, belonged to none of the incompatibility types, had a molecular weight of 8–11 kpb and encoded *mphA*.

**Figure 4:**
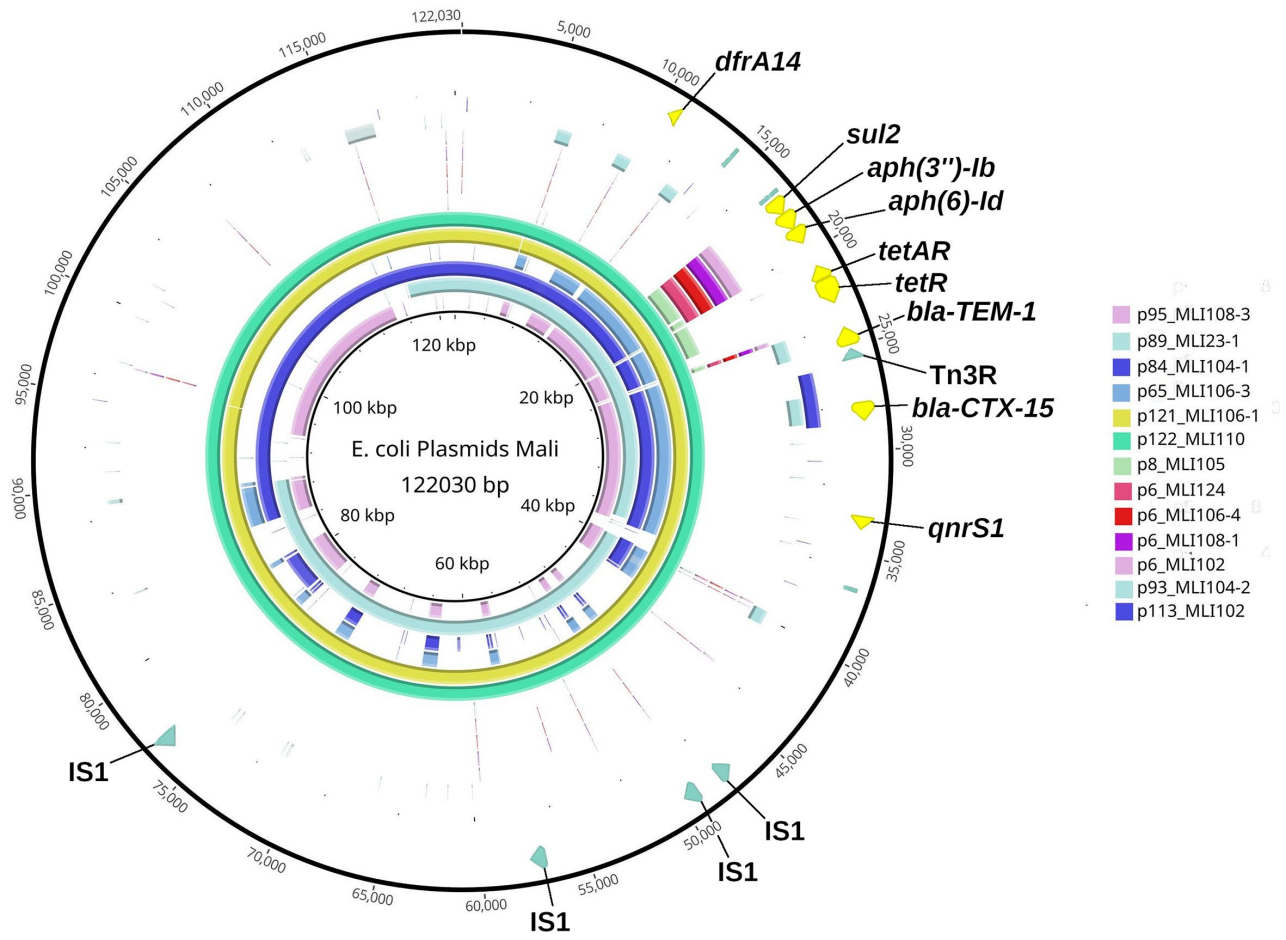
Blast Ring Image Generator-(BRIG-)generated graph depicting homologous plasmid segments of the plasmid types 2–4 detected in the *Escherichia coli* isolates from soldiers probed in Mali. The parameters used to generate the BRIG-graph were: BLAST type: blastn, upper identity threshold 70%, lower identity threshold 30%, ring size 30, and plasmid origin at 12 o’clock position. The largest plasmid of these three plasmid types, type 2 plasmid p122_MLI110, was chosen as reference (inner black circle) and is additionally shown in the sixth outer circle (mint-green). The individual plasmids are color coded as in the legend and the ARGs are annotated. Outermost Ring: plasmid-map of p122_MLI110 indicating the particular ARGs in yellow and specific MGE recognition sequences in olive green.

From the heat map in Figure 3, it can be already assumed that most ARGs of plasmid types 2, 3, and 4 are located on segments of different sizes with higher sequence identity. All six plasmids of type 2 share a plasmid segment that encodes the ARGs *dfrA14*, *sul2*, *aph*(*3″*) *− Ib*, *aph*(*6*) *− Id*, *tetA*/*tetR*, *bla*_TEM − 1_, *bla*_CTX − M − 15_, and *qnrS1* (Figure 4). A subsegment containing the ARGs *sul2*, *aph*(*3″*) *−* (*Ib*), *and aph*(*6*) *− Id* was found in the five type 3 plasmids. Another subsegment encoding *bla*_CTX-M-15_ existed in the two type 4 plasmids. A subsegment encoding *bla*_TEM-1_ was found on the type 4 plasmid p93_MLI104-2, as well as on the type 2 plasmids. These (sub-) segments were most likely transferred together at one point of plasmid evolution on different mobile genetic elements, so that in particular the type 2 plasmids can be conceived as plasmid chimeras. As an example, we will take a closer look at the type 2 plasmid p122_MLI110. At position 15.208–63.996 there is a composite transposon, cn_48788_IS5075 (family IS110), encoding the ARGs *sul2*, *aph*(*3″*) *− Ib*, *aph*(*6*) *− Id*, *tetA*/*tetR*, *bla*_TEM − 1_, *bla*_CTX − M − 15_, and *qnrS1*. A second composite transposon, cn_27847_IS26 (family IS6), at position 12.611–40.458, covers the same ARGs. ARG *dfrA14* is on a separate MGE, cn_4021_IS26 (family IS6) at position 9.410–13.431. In contrast, there was only one MGE on the type 4 plasmid p93_MLI104-2. While the composite transposon, cn_3146_IS26, at position 87.935–91.081, carried *bla*_CTX − M − 15_, the two ARGs *bla*_TEM − 1_ and *aac*(*3*)*-IId* were encoded on the remaining plasmid regions. The type 3 plasmid p6_MLI124, encoding *sul2*, *aph*(*3″*) *− Ib*, and *aph*(*6*) *− Id*, carried no MGE. Mobile genetic elements of all chromosomes and plasmids associated with antimicrobial resistance genes are listed in the Supplementary Table 1.

### 3.5. Identified virulence determinants

Recorded virulence factors mostly comprised genes affecting the bacterial iron metabolism, while less frequently observed genes influenced mechanisms like capsule formation and the excretion of substances from the cell. Details are provided in the Supplementary Table 2. Only in a single instance (MLI109) and thus well in line with a previous observation (Frickmann et al., 2015), the presence of the *aggR* gene confirmed the isolate as enteroaggregative *Escherichia coli* and thus as an isolate with enteropathogenic potential. The *E. coli* isolate MLI 109 carries one virulence plasmid on which the *aggR* gene was localized, which showed in a BLAST search a sequence identity >99% to pAA plasmids of other *E. coli* isolates deposited at NCBI GenBank. Consequently, the *E. coli* isolate MLI 109 is a typical representative of the EAEC (Enteroaggregative ***E**scherichia **c**oli*) pathovar.

## 4. Discussion

In this study, we characterized 19 isolates (morphotypes) of third-generation cephalosporin resistant *Escherichia coli* from diarrhea stool samples of 12 European soldiers during the EUTM (European Training Mission) Mali mission between December 2013 and August 2014 (Frickmann et al., 2015; Hagen et al., 2015). Based on the results, the following conclusions appear justified.

First, a high molecular diversity of the isolates from the different individuals was confirmed, similar as suggested by rep-typing in a previous analysis (Frickmann et al., 2015). Only in two cases, a transmission from soldier to soldier or from common sources could be demonstrated. These results suggest that direct transmission between the different soldiers or transmission from a common source were just exceptions. The high genetic diversity of the isolates suggests multiple transmission events, most likely reflecting a high local prevalence of different ESBL-positive *E. coli* strains. This is underscored by the fact that genetically heterogenic third-generation cephalosporin-resistant *E. coli* isolates were found in stool samples from a single individual. Antibiotic exposure seemed to have played—if any—only a minor role in the acquisition of ESBL-positive *E. coli* by the assessed European soldiers suffering from diarrhea on tropical deployment in contrast to previous suggestions (Kantele et al., 2015). Therefore, no respective details have been presented. In short and as recorded for the previously described epidemiological assessment (Frickmann et al., 2015), only the patient from whom the isolate MLI-107 was isolated received treatment of traveler’s diarrhea with rifaximin, while malaria prophylaxis with 100 mg once daily doxycycline was taken by the patients providing the isolates MLI-104_1 and MLI-102_2 as well as MLI-108_1, MLI-108_2, and MLI-108_3. It may be speculated that the low-dose doxycycline application might have triggered the selection of MLI-104_1 and MLI-108_3 *via* the co-existing *tet*(*A*)-gene and the selection of MLI_104_2 *via* the co-existing *tet*(*D*)-gene, however, the extent of this effect remains speculative.

Second, next-generation sequencing analyses allowed a deeper insight into the molecular background of the recorded third-generation cephalosporin resistance than a previous multiplex PCR- and Sanger sequencing-based approach (Hagen et al., 2015). The ESBL-mediating gene *bla*_CTX-M-15_ [with (*n* = 14/19) or without (*n* = 5/19) *bla*_TEM-1b_] was detected in all isolates without exemption. This finding is expected, since *bla*_CTX-M-15_ is known to be highly prevalent in multiple countries of Sub-Saharan Africa (Mshana et al., 2013; Onduru et al., 2021). Interestingly, both chromosomal and plasmid-encoded *bla*_CTX-M-15_ genes were detected in the isolates, whereas a study in neighboring Ghana found *bla*_CTX-M-15_ encoded exclusively on plasmids in the *E. coli* isolates examined (Pankok et al., 2022b).

Third, the assessment of the sequence types did not indicate known pathogenic *E. coli* clonal lineages (Denamur et al., 2021) and in line with this, few virulence factors mediating enteropathogenicity were documented. Only one isolate was confirmed as enteroaggregative *E. coli* as shown in a previous publication (Frickmann et al., 2015), however, the etiological relevance of *E. coli* harboring this virulence determinant has been challenged recently (Tisdale et al., 2022).

In addition, the analysis provided a deeper insight into locally abundant resistance gene-carrying plasmids as well as resistance genes mediating resistance to antimicrobial drugs other than third-generation cephalosporines. Thus, we were able to categorize the observed plasmids into five types, which, in turn, share different sequence-identical segments carrying specific ARGs. A broad variety of plasmid-encoded and chromosomal resistance genes as well as antimicrobial resistance gene-associated mobile genetic elements was detected, explaining the observed resistance against other none-beta-lactam drugs like fluoroquinolones, aminoglycosides, epoxide antibiotics (fosfomycin), and trimethoprim-sulfamethoxazole commonly used for the therapy of infections with Enterobacterales as well. This information contributes to the scarcely available data on AMR in Gram-negative bacteria in Mali.

Of note, occurrence of variable *in vitro* susceptibility toward piperacillin-tazobactam rather than general *in-vitro* resistance in spite of *bla*_CTX-M-15_-carriage is the rule and not the exemption as repeatedly shown in international literature (Bradford, 2001; Titelman et al., 2011; Rosenkilde et al., 2019). This is also true for the observed only partial correlation of cotrimoxazole resistance with the observed resistance mediating genes. The *dfrA* gene group encodes trimethoprim-insensitive homologs of the bacterial dihydrofolate reductase with varying levels of residual trimethoprim susceptibility (Lombardo et al., 2016; Ambrose and Hall, 2021). The common *dfrA1* gene, for example, has been associated with 1,000-fold reduced susceptibility toward trimethoprim (Lombardo et al., 2016). The also observed genes *sul1* and *sul2* encode variants of the dihydropteroate synthase in the folic acid pathway mediating insensitivity toward sulfonamides (Sköld, 2000). Of note, however, *sul* genes have been reported to show a tremendous variance of expression in *E. coli* as a fitness cost compensation mechanism, a phenomenon, which is likely to be associated with the observed variance of cotrimoxazole resistance in this study (Zhou et al., 2021).

The study has a number of limitations. First, we analyzed a small number of isolates in depth, which limits representativeness. Second, the samples were collected several years ago and may not reflect strains currently occurring in Mali. Nevertheless, the sequences of the studied isolates may be useful as reference in AMR surveillance. Third, since there was no initial screening for ESBL carriage, we cannot exclude completely that some isolates were imported by the deployed soldiers. However, frequent personnel turnover is typical for a military camp. There are several factors speaking in favor of the hypothesis that at least a considerable proportion of ESBL-positive *E. coli* had been acquired on deployment in Mali. The additional cgMLST-based relationship analysis we performed showed, with the exception of only one isolate (MLI124), an arrangement of the isolates from Mali with isolates from Niger and Ghana in a supercluster that supported their West African origin as probable. Within the time period when the assessed isolates had been collected, the civilian study group of Lübbert and colleagues reported enteric colonization with ESBL-positive Enterobacterales in about one out of three travel returnees from Western Africa, while the average prevalence in the European standard population was considerably lower at this time (Lübbert et al., 2015). The prevalence within the screened soldier population matched this ESBL prevalence of the returnee’s in Lübbert’s study nearly exactly, suggesting a comparable underlying epidemiological background. While we are well aware that the details of individual spatial–temporal acquisition of each isolate cannot be defined with certainty and even comparisons with sequences deposited in available databases might be misleading, because the isolation site of a bacterium is not necessarily identical with the initial transmission site, we nevertheless feel justified by the abovementioned epidemiological considerations to assume a Malian origin of at least a major proportion of the assessed isolates. The practical relevance of such estimations remains nevertheless debatable, because transmission events can occur in both directions and so, mutual transmission events both from Malian individuals to European soldiers and vice versa have to be assumed and the survival of the transmitted strains in the respective populations necessarily depended on the individual strain’s evolutionary fitness.

## 5. Conclusion

With this study, we extended the knowledge on the molecular epidemiology of AMR in Western African Mali and detected a high local prevalence of *bla*_CTX-M-15_. This underscores the need for interventions that tackle the worldwide problem of AMR.

## Data availability statement

The datasets presented in this study can be found in online repositories. The names of the repository/repositories and accession number(s) can be found at: https://www.ncbi.nlm.nih.gov/genbank/, CP116913; https://www.ncbi.nlm.nih.gov/genbank/, CP116911; https://www.ncbi.nlm.nih.gov/genbank/, CP116912; https://www.ncbi.nlm.nih.gov/genbank/, CP116916; https://www.ncbi.nlm.nih.gov/genbank/, CP116914; https://www.ncbi.nlm.nih.gov/genbank/, CP116915; https://www.ncbi.nlm.nih.gov/genbank/, CP116919; https://www.ncbi.nlm.nih.gov/genbank/, CP116917; https://www.ncbi.nlm.nih.gov/genbank/, CP116918; https://www.ncbi.nlm.nih.gov/genbank/, CP116923; https://www.ncbi.nlm.nih.gov/genbank/, CP116920; https://www.ncbi.nlm.nih.gov/genbank/, CP116921; https://www.ncbi.nlm.nih.gov/genbank/, CP116922; https://www.ncbi.nlm.nih.gov/genbank/, CP116925; https://www.ncbi.nlm.nih.gov/genbank/, CP116924; https://www.ncbi.nlm.nih.gov/genbank/, CP116929; https://www.ncbi.nlm.nih.gov/genbank/, CP116926; https://www.ncbi.nlm.nih.gov/genbank/, CP116927; https://www.ncbi.nlm.nih.gov/genbank/, CP116928; https://www.ncbi.nlm.nih.gov/genbank/, CP116931; https://www.ncbi.nlm.nih.gov/genbank/, CP116930; https://www.ncbi.nlm.nih.gov/genbank/, CP117049; https://www.ncbi.nlm.nih.gov/genbank/, CP117046; https://www.ncbi.nlm.nih.gov/genbank/, CP117047; https://www.ncbi.nlm.nih.gov/genbank/, CP117048; https://www.ncbi.nlm.nih.gov/genbank/, CP117020; https://www.ncbi.nlm.nih.gov/genbank/, CP117017; https://www.ncbi.nlm.nih.gov/genbank/, CP117018; https://www.ncbi.nlm.nih.gov/genbank/, CP117019; https://www.ncbi.nlm.nih.gov/genbank/, CP117053; https://www.ncbi.nlm.nih.gov/genbank/, CP117050; https://www.ncbi.nlm.nih.gov/genbank/, CP117051; https://www.ncbi.nlm.nih.gov/genbank/, CP117052; https://www.ncbi.nlm.nih.gov/genbank/, CP116987; https://www.ncbi.nlm.nih.gov/genbank/, CP116994; https://www.ncbi.nlm.nih.gov/genbank/, CP116988; https://www.ncbi.nlm.nih.gov/genbank/, CP116989; https://www.ncbi.nlm.nih.gov/genbank/, CP116990; https://www.ncbi.nlm.nih.gov/genbank/, CP116991; https://www.ncbi.nlm.nih.gov/genbank/, CP116992; https://www.ncbi.nlm.nih.gov/genbank/, CP116993; https://www.ncbi.nlm.nih.gov/genbank/, CP117001; https://www.ncbi.nlm.nih.gov/genbank/, CP116995; https://www.ncbi.nlm.nih.gov/genbank/, CP116996; https://www.ncbi.nlm.nih.gov/genbank/, CP116997; https://www.ncbi.nlm.nih.gov/genbank/, CP116998; https://www.ncbi.nlm.nih.gov/genbank/, CP116999; https://www.ncbi.nlm.nih.gov/genbank/, CP117000; https://www.ncbi.nlm.nih.gov/genbank/, CP117006; https://www.ncbi.nlm.nih.gov/genbank/, CP117002; https://www.ncbi.nlm.nih.gov/genbank/, CP117003; https://www.ncbi.nlm.nih.gov/genbank/, CP117004; https://www.ncbi.nlm.nih.gov/genbank/, CP117005; https://www.ncbi.nlm.nih.gov/genbank/, CP117008; https://www.ncbi.nlm.nih.gov/genbank/, CP117007; https://www.ncbi.nlm.nih.gov/genbank/, CP117010; https://www.ncbi.nlm.nih.gov/genbank/, CP117009; https://www.ncbi.nlm.nih.gov/genbank/, CP117013; https://www.ncbi.nlm.nih.gov/genbank/, CP117011; https://www.ncbi.nlm.nih.gov/genbank/, CP117012; https://www.ncbi.nlm.nih.gov/genbank/, CP117016; https://www.ncbi.nlm.nih.gov/genbank/, CP117014; https://www.ncbi.nlm.nih.gov/genbank/, CP117015; https://www.ncbi.nlm.nih.gov/genbank/, JANLNI000000000; https://www.ncbi.nlm.nih.gov/genbank/, JANLNI010000027.1; https://www.ncbi.nlm.nih.gov/genbank/, JANLNI010000050.1; and https://www.ncbi.nlm.nih.gov/genbank/, JANLNI010000064.1.

## Author contributions

HF, RH, and AZ: conceptualization. AT, PM, MR, and SK: methodology. MR: software. MR, KH, SK, and AZ: validation. MR, KH, and AZ: formal analysis. KH, RH, and AZ: investigation. HF, AZ, and AK: resources. MR, KH, AZ, and HF: data curation. HF and AZ: writing—original draft preparation, supervision, and project administration. KH, MR, PM, AT, SK, AK, HF, RH, and AZ: writing—review and editing. MR and KH: visualization. AK: funding acquisition. All authors contributed to the article and approved the submitted version.

## Funding

This work was funded by the Deutsche Forschungsgemeinschaft (DFG; grant number ZA 697/6–1).

## Conflict of interest

The authors declare that the research was conducted in the absence of any commercial or financial relationships that could be construed as a potential conflict of interest.

## Publisher’s note

All claims expressed in this article are solely those of the authors and do not necessarily represent those of their affiliated organizations, or those of the publisher, the editors and the reviewers. Any product that may be evaluated in this article, or claim that may be made by its manufacturer, is not guaranteed or endorsed by the publisher.

## Footnotes

^1^ https://github.com/lh3/miniasm

^2^ https://github.com/xiaochuanle/NECAT

^3^ https://github.com/lbcb-sci/raven

^4^ https://github.com/nanoporetech/medaka

^5^ https://github.com/rrwick/Polypolish
