# Supplementary material for: Molecular epidemiology of enterically colonizing Escherichia coli with resistance against third-generation cephalosporins isolated from stool samples of European soldiers with concomitant diarrhea on deployment in Western African Mali
Source: Front Microbiol. 2023 May 5;14:1169829. doi: 10.3389/fmicb.2023.1169829 (PMC10198576; doi:10.3389/fmicb.2023.1169829)

Supplementary Material

Molecular epidemiology of *Escherichia coli* with resistance against third-generation cephalosporins isolated from stool samples of European soldiers with diarrhea on deployment in Western African Mali

Katharina Hoffmann, Matthias Riediger, Aljoscha Tersteegen, Pauline Marquardt, Sascha Kahlfuß, Achim J. Kaasch, Ralf Matthias Hagen, Hagen Frickmann and Andreas E. Zautner*

*** Correspondence:** Andreas E. Zautner: azautne@gwdg.de

1 Supplementary Tables

Supplementary table 1: Clonal lineages, identified plasmid types and recorded antimicrobial resistance genes (ARG) as well as ARG-associated genetic elements (MGE) of the assessed *Escherichia coli* isolates with resistance against 3rd generation cephalosporines (based on the software Resfinder, MGEFinder and Plasmid-Finder). Underlined print marks 3rd generation cephalosporine resistance-associated genes.

Sample I.D. / BioSample accession number	Sequence types according to Achtmann (a) / Pasteur (p)	Chromosome and recorded plasmid types (Inc-group, GenBank accession)	Antimicrobial resistance genes, (s) results by SNP pointfinder, (f) results by resfinder functional genomics, (r) results by armfinderplus	ARG-associated genetic elements (MGE)
MLI23-1/ SAMN29553880	ST-8149[a] / ST-698[p]	chromosome (none, CP116913)	*van_ligase*[f], *dfr*[f], *beta_lactamaseEC*[f], *non_enz_beta_lactam_resistance*[f], *mdtM*[r], *acrF*[r], *emrD*[r]	MITEEc1,ISEc1,ISEc5, ISKpn8, ISKpn26, ISKpn37, IS3, IS30, IS421, IS609
		p89_MLI23-1 (IncFIB, CP116911)	*aph(3'')-Ib, aph(6)-Id, sul2, tet(A), qnrS1, dfrA14,* bla_{TEM-1B}, $bla_{CTX-M-15}$, *dfr*[f], *tet_efflux*[f], *dpr*[f] *beta_lactamase*[f]	IS5, IS26, IS5075, ISKpn19, ISKpn26,

		p35_MLI23-1 (none, CP116912)	none	none
MLI23-2/ SAMN29553881	ST-8149[a] / ST-698[p]	chromosome (none, CP116916)	*van_ligase*[f], *dfr*[f], *beta_lactamaseEC*[f], *non_enz_beta_lactam_resistance*[f], *mdtM*[r], *acrF*[r], *emrD*[r]	MITEEc1, ISEc1, ISEc5, ISKpn8, ISKpn26, ISKpn37, IS3, IS30, IS421, IS609
		p89_MLI23-2 (IncFIB, CP116914)	*aph(3'')-Ib, aph(6)-Id, sul2, tet(A), qnrS1, dfrA14, bla*$_{TEM-1B}$, *bla*$_{CTX-M-15}$, *dfr*[f], *tet_efflux*[f], *dpr*[f] *beta_lactamase*[f]	IS5, IS26, IS5075, ISKpn19, ISKpn26,
		p35_MLI23-2 (none, CP116915)	none	none
MLI102/ SAMN29553882	ST-38[a]/ ST-8[p]	chromosome (none, CP116919)	*gyrA*[s], *aadA1*, *gyrA:p.S83L, tet(B), bla*$_{OXA-1}$, *catA1*,*tet_efflux*[f],*van_ligase*[f],*beta_lactamaseEC*[f], *non_enz_beta_lactam_resistance*[f], *cat*[f], *mdtM*[r], *acrF*[r], *emrD*[r]	IS3, IS5, IS421, IS629, IS911, MITEEc1, ISEc1, , ISEc40, ISEc78, ISSfl8, ISSfl10, , ISSpu2
		p113_MLI102 (none, CP116917)	*bla*$_{CTX-M-15}$, *beta_lactamase*[f]	ISEc9, IS421
		p6_MLI102 (none, CP116918)	*aph(3'')-Ib, aph(6)-Id, sul2, dpr*[f],	none
MLI104-1/ SAMN29553883	ST-10[a] / ST-novel[p]	chromosome (none, CP116923)	*van_ligase*[f], *dfr*[f], *beta_lactamaseEC*[f], *non_enz_beta_lactam_resistance*[f], *mdtM*[r], *emrD*[r]	MITEEc1, IS3, IS100, IS102, IS421, IS609, ISEc1, ISEc5, ISEc19, ISEsa1, ISKpn8
		p84_MLI104-1 (IncFIB, CP116920)	*aph(3'')-Ib, aph(6)-Id, qnrS1, dfrA14, sul2, bla*$_{TEM-1B}$, *bla*$_{CTX-M-15}$, *tet(A), tet_efflux*[f], *beta_lactamase*[f], *dfr*[f], *dpr*[f]	ISEc9, ISKpn19, ISKox3, IS26, IS5075
		p3_MLi104-1 (none, CP116921)	none	ISEc30
		p2_MLi104-1(none, CP116922)	none	none

MLI104-2/ SAMN29553884	ST-10[a] / ST-367[p]	chromosome (none, CP116925)	*aadA1, dfrA1, sul1, catA1*, *tet(D)*, qacE, *dpr*[f], *van_ligase*[f], *beta_lactamaseEC*[f], *dfr*[f], *cat*[f], *non_enz_beta_lactam_resistance*[f], *tet_efflux*[f], *mdtM*[r], *acrF*[r], *emrD*[r]	IS3, IS4, IS5, IS26, IS102, IS421, IS609, IS629, IS682, ISEc1, ISEc5, ISEc38, ISEc45, ISEc78, ISEsa1, MITEEc1, ISSfl8, ISSfl10, ISKpn26
		p93_MLI104-2 (IncI1, CP116924)	*aac(3)-IId*, bla_{TEM-1B}, $bla_{CTX-M-15}$, *beta_lactamase*[f], *AAC*[f]	IS26
MLI105/ SAMN29553885	ST-10[a] / ST-2[p]	chromosome (none, CP116929)	*aadA1, gyrA:p.S83A, dfrA1*, $bla_{CTX-M-15}$, *gyrA*[s], *beta_lactamaseEC*[f],*van_ligase*[f], *non_enz_beta_lactam_resistance*[f], *dfr*[f], *mdtM*[r], *acrF*[r], *emrD*[r], *sat2*[r]	ISEc1, ISEc5, ISEc9, IS3, IS4, IS26, IS30, IS100, IS102, IS421, IS609, IS911, ISKpn8, ISKpn24, MITEEc1, ISSfl10
		p11_MLI105 (none, CP116926)	*mph(A)*, bla_{TEM-1B}, *beta_lactamase*[f]	IS6100
		p8_MLI105 (none, CP116927)	*tet(A), sul2, aph(3'')-Ib, aph(6)-Id, tet_efflux*[f], *dpr*[f]	none
		p5_MLi105 (none, CP116928)	none	none
MLI106-1/ SAMN29553886	ST-38[a] / ST-535[p]	chromosome (none, CP116931)	*sitABCD, van_ligase*[f], *beta_lactamaseEC*[f], *non_enz_beta_lactam_resistance*[f], *mdtM*[r], *acrF*[r], *emrD*[r]	MITEEc1, ISEc1, ISEc30, ISEc31, ISEc38, ISEc46, IS4, IS609, IS911, ISKpn8, ISSfl10,
		p121_MLI106-1 (IncFIB, CP116930)	*aph(3'')-Ib, aph(6)-Id, qnrS1, sul2, dfrA14*, $bla_{CTX-M-15}$, bla_{TEM-1B}, *tet(A), beta_lactamase*[f], *tet_efflux*[f], *dhr*[f], *dpr*[f]	ISEc9, ISKpn19, IS5, IS26, IS5075, ISKox3
MLI106-2/ SAMN29553887	ST-617[a] / ST-2[p]	chromosome (none, CP117049)	*parE:p.S458A, gyrA:p.S83L, parC:p.S80I, gyrA:p.D87N, parE*[s], *parC*[s],*gyrA*[s], *van_ligase*[f], *non_enz_beta_lactam_resistance*[f], *dfr*[f], *beta_lactamaseEC*[f],	MITEEc1, IS3, IS4, IS5, IS26, IS30, IS100, IS102, IS421, IS609, IS629, IS911, ISEc1,

			mdtM[r], *acrF*[r], *emrD*[r]	ISEc5, ISKpn8, ISKpn24, ISKpn26, ISKox3,
		p174_MLI106-2(IncFII, IncFIB, IncFIA, CP117046)	*aadA5, aac(3)-IId, aac(6')-Ib-cr, mph(A), sul1, dfrA17, sitABCD, tet(B),*bla_{TEM-1B}, bla_{OXA-1}, $bla_{CTX-M-15}$, *qacE, catB3, catA1, AAC*[f], *beta_lactamase*[f], *cat*[f], *tet_efflux*[f] , *dpr*[f]	IS26, IS100, IS6100, ISEc9, ISKpn8, ISKpn24
		p6_MLI106-2 (none, CP117047)	*aph(3'')-Ib, aph(6)-Id, sul2, dpr*[f]	none
		p4_MLI106-2 (none, CP117048)	none	none
MLI106-3/ SAMN29553888	ST-38[a] / ST-535[p]	chromosome (none, CP117020)	*van_ligase*[f], *non_enz_beta_lactam_resistance*[f], *beta_lactamaseEC*[f], *mdtM*[r], *acrF*[r], *emrD*[r]	IS4, IS100, IS609, IS911, MITEEc1, ISKpn8, ISEc1, ISEc46, ISEc81, ISSfl10
		p65_MLI106-3 (IncY, CP117017)	*tet(A), qnrS1, sul2, dfrA14,* bla_{TEM-1B}, $bla_{CTX-M-15}$, *aph(3'')-Ib, aph(6)-Id, beta_lactamase*[f], *dpr*[f], *dfr*[f] , *tet_efflux*[f]	ISEc9, ISKpn19, IS26
		p3_MLI106-3 (none, CP117018)	none	none
		p1_MLI106-3 (none, CP117019)	none	none
MLI106-4/ SAMN29553889	ST-617[a] / ST-2[p]	chromosome (none, CP117053)	*parE:p.S458A, gyrA:p.S83L, parC:p.S80I, gyrA:p.D87N, parE*[s], *parC*[s], *gyrA*[s], *van_ligase*[f], *non_enz_beta_lactam_resistance*[f], *dfr*[f], *beta_lactamaseEC*[f], *mdtM*[r], *acrF*[r], *emrD*[r]	MITEEc1, IS3, IS4, IS5, IS26, IS30, IS100, IS102, IS421, IS609, IS629, IS911, ISEc1, ISEc5, ISKpn8, ISKpn24, ISKpn26, ISKox3,
		p174_MLI106-4 (IncFIA,IncFII, IncFIB, CP117050)	*aadA5, aac(3)-IId, aac(6')-Ib-cr, mph(A), sul1, dfrA17, sitABCD, tet(B),*bla_{TEM-1B}, bla_{OXA-1}, $bla_{CTX-M-15}$, *qacE, catB3, catA1,* *AAC*[f], *beta_lactamase*[f], *cat*[f], *tet_efflux*[f] , *dpr*[f]	IS26, IS100, IS6100, ISEc9, ISKpn8, ISKpn24

		p6_MLI106-4 (none, CP117051)	*aph(6)-Id, aph(3'')-Ib, sul2, dpr*[f]	none
		p4_MLI106-4 (none, CP117052)	none	none
MLI107/ SAMN29553890	ST-4681[a] / ST-355[p]	chromosome (none, CP116987)	*$bla_{CTX-M-15}$, van_ligase*[f], *dfr*[f], *beta_lactamaseEC*[f], *non_enz_beta_lactam_resistance*[f], *mdtM*[r], *acrF*[r], *emrD*[r]	ISEc9, MITEEc1, IS609, IS621, ISEc1
MLI108-1/ SAMN29553891	ST-44[a] / ST-2[p]	chromosome (none, CP116994)	*gyrA:p.D87N, parC:p.S80I, parE:p.S458T, gyrA:p.S83L, parE*[s], *parC*[s], *gyrA*[s], *dfr*[f], *non_enz_beta_lactam_resistance*[f], *beta_lactamaseEC*[f], *van_ligase*[f], *mdtM*[r], *acrF*[r], *emrD*[r]	MITEEc1, ISEc1, ISEc5, ISEc31, IS3, IS26, IS30, IS100, IS102, IS421, IS609, IS621, ISKpn8, ISKpn24, ISKox3,
		p172_MLI108-1 (IncFIA,IncFII,IncFIB, CP116988)	*dfrA17, sul1, aac(6')-Ib-cr, aadA5, aac(3)-IIa, mph(A), sitABCD, tet(B), $bla_{CTX-M-15}$, bla_{OXA-1}, qacE, catB3, beta_lactamase*[f], *AAC*[f], *tet_efflux*[f], *dpr*[f], *cat*[f]	IS26, IS100, IS5075, Tn5403, ISKpn8, ISKox3
		p92_MLI108-1 (none, CP116989)	none	IS609
		p12_MLI108-1 (none, CP116990)	none	none
		p6_MLI108-1 (none, CP116991)	*aph(3'')-Ib, aph(6)-Id, sul2, dpr*[f]	none
		p3_MLI108-1 (none, CP116992)	none	none
		p1_MLI108-1 (none, CP116993)	none	none
MLI108-2/ SAMN29553892	ST-44[a] / ST-2[p]	chromosome (none, CP117001)	*gyrA:p.D87N, parC:p.S80I, parE:p.S458T, gyrA:p.S83L, parE*[s], *parC*[s], *gyrA*[s], *dfr*[f], *non_enz_beta_lactam_resistance*[f], *beta_lactamaseEC*[f], *van_ligase*[f], *mdtM*[r], *acrF*[r], *emrD*[r]	MITEEc1, ISEc1, ISEc5, ISEc31, IS3, IS26, IS30, IS100, IS102, IS421, IS609, IS621, ISKpn8, ISKpn24, ISKox3,

		p172_MLI108-2 (IncFIA,IncFII , IncFIB, CP116995)	*dfrA17, sul1, aac(6')-Ib-cr, aadA5, aac(3)-IIa, mph(A), sitABCD, tet(B),* $bla_{CTX-M-15}$*,* bla_{OXA-1}*, qacE, catB3, beta_lactamase*[f]*, AAC*[f]*, tet_efflux*[f]*, dpr*[f]*, cat*[f]	IS26, IS100, IS5075, Tn5403, ISKpn8, ISKox3
		p92_MLI108-2 (none, CP116996)	none	IS609
		p12_MLI108-2 (none, CP116997)	none	none
		p6_MLI108-2 (none, CP116998)	*aph(3'')-Ib, aph(6)-Id, sul2, dpr*[f]	none
		p3_MLI108-2 (none, CP116999)	none	none
		p1_MLI108-2 (none, CP117000)	none	none
MLI108-3/ SAMN29553893	ST-3268[a] / ST-535[p]	chromosome (none, CP117006)	*sitABCD, van_ligase*[f]*, beta_lactamaseEC*[f]*, non_enz_beta_lactam_resistance*[f]*, mdtM*[r]*, acrF*[r]*, emrD*[r]	ISKpn8,IS4,IS609, ISEc1,ISEc17, ISEc31,ISEc38, ISEc46, MITEEc1
		p108_MLI108-3 (none, CP117002)	none	none
		p95_MLI108-3 (IncY, CP117003)	*tet(A), qnrS1, aph(3'')-Ib, aph(6)-Id, dfrA14, sul2,* $bla_{CTX-M-15}$*,* bla_{TEM-1B}*, beta_lactamase*[f]*, tet_efflux*[f]*, dpr*[f]*, dfr*[f]	ISEc9, IS4, IS26, IS5075, ISKpn19
		p89_MLI108-3 (IncFIC,IncFIB,CP117004)	none	IS30, IS5075
		p4_MLI108-3(none, CP117005)	none	none
MLI109/ SAMN29553894	ST-38[a] / ST-8[p]	chromosome (none, CP117008)	*aph(3'')-Ib, aadA1, aph(6)-Id, aac(6')-Ib-cr, aac(3)-IIa, gyrA:p.S83L, dfrA1, tet(A), tet(D), sul2,* bla_{TEM-1B} *.* $bla_{CTX-M-15}$*, blaOXA-1, catB3, catA1,gyrA*[s]*, van_ligase*[f]*, beta_lactamaseEC*[f]*,*	ISVsa3, IS26, IS609, IS5075, ISEc1, ISEc9, ISEc38, ISEc46, ISKpn8, MITEEc1,

			AAC[f], *tet_efflux*[f], *cat*[f], *non_enz_beta_lactam_resistance*[f], , *dpr*[f] ,*acrF*[r], *emrD*[r], *sat2*[r]	Tn7, Tn5403,
		p140_MLI109 (IncFIB,IncFII, CP117007)	none	ISEc31, ISEc38, ISSpu2, IS3, IS629
MLI110/ SAMN29553895	ST-48[a] / ST-novel[p]	chromosome (none, CP117010)	*beta_lactamaseEC*[f], *van_ligase*[f], *dfr*[f], *non_enz_beta_lactam_resistance*[f], *mdtM*[r], *acrF*[r], *emrD*[r]	MITEEc1, ISKpn8, ISKpn24, ISEc1, ISEc5, ISEc38, IS3, IS5, IS30, IS421, IS609, IS629,
		p122_MLI110 (IncFIB, CP117009)	*tet(A), qnrS1, dfrA14, sul2,* $bla_{CTX-M-15}$, bla_{TEM-1B}, *aph(3'')-Ib, aph(6)-Id, beta_lactamase*[f], *tet_efflux*[f], *dfr*[f], *dpr*[f]	ISEc9, ISKpn19, ISKox3, IS5, IS26, IS5075
MLI114/ SAMN29553896	ST-349[a] / ST-678[p]	chromosome (none, CP117013)	*gyrA:p.S83L, sul1, dfrA5, tet(A), qacE ,* bla_{TEM-1B}, $bla_{CTX-M-15}$, *gyrA*[s] ,*van_ligase*[f], *beta_lactamaseEC*[f], *non_enz_beta_lactam_resistance*[f], *tet_efflux*[f] ,*dpr*[f] , *mdtM*[r], *acrF*[r], *emrD*[r]	IS30, IS609, IS629, MITEEc1,ISKpn8, ISKpn24, ISSfl10, ISEc1, ISEc9, ISEc10, ISEc18, ISEc38, ISEc45, ISEc46
		p80_MLI114 (IncI, CP117011)	none	none
		p8_MLI114 (none, CP117012)	*mph(A)*	IS26
MLI121/ SAMN29553897	ST-349[a] / ST-678[p]	chromosome (none, CP117016)	*gyrA*:p.S83L, *dfrA5, sul1, tet(A),* bla_{TEM-1B}. $bla_{CTX-M-15}$, *qacE, gyrA*[s], *van_ligase*[f], *tet_efflux*[f] *non_enz_beta_lactam_resistance*[f], *beta_lactamaseEC*[f], *dpr*[f] ,*mdtM*[r], *acrF*[r], *emrD*[r]	IS26, IS30, IS609, IS629, ISKpn8, ISKpn24, ISEc1, ISEc9, ISEc10, ISEc18, ISEc38, ISEc45, ISEc46, MITEEc1, ISSfl10
		p95_MLI121 (none, CP117014)	none	none
		p8_MLI121 (none, CP117015)	*mph(A)*	IS26

MLI124[*]/ SAMN29553898	ST-940[a] / ST-866[p]	chromosome (JANLNI000000000)	*tet(B), gyrA:p.S83L, dfrA1,* $bla_{CTX\text{-}M\text{-}15}$, *gyrA*[s], *beta_lactamaseEC*[f], *van_ligase*[f], *non_enz_beta_lactam_resistance*[f], *tet_efflux*[f] ,*mdtM*[r], *acrF*[r], *emrD*[r], *sat2*[r]	IS3, IS30, ISEc1, ISEc9, MITEEc1, IS609, IS621, IS629, IS911,
		p68_MLI124 (IncB/O/K/Z, JANLNI010000027.1)	none	ISEc37, IS629
		p15_MLI124 (IncFII, JANLNI010000050.1)	none	ISCfr13, ISSfl10, IS629
		p6_MLI124 (none, JANLNI010000064.1)	*aph(6)-Id, aph(3'')-Ib, sul2, dpr*[f]	none

[*]Due to technical reasons it was not possible to close the chromosome of *E. coli* MLI124. The genome coverage of the Illumina sequencing was 57.2%. For the closed plasmids the GenBank accesion numbers of the contigs are given.

Supplementary table 2: Virulence factors identified in the isolates. The *aggR* gene, indicating enteroaggregative *E. coli*, was detected in the MLI109 isolate only.

Sample I.D.	Genes encoding Virulence associated factors
MLI23-1	*fyuA* - Siderophore receptor
	irp2 - High molecular weight protein 2 non-ribosomal peptide synthetase
	iss - Increased serum survival
	terC - Tellurium ion resistance protein
	csgA - curlin major subunit CsgA
	fdeC - intimin-like adhesin FdeC
	gad - Glutamate decarboxylase
	hlyE - Avian E. coli haemolysin
	nlpI - lipoprotein NlpI precursor

	(plasmid none)
MLI23-2	*fyuA* - Siderophore receptor
	irp2 - High molecular weight protein 2 non-ribosomal peptide synthetase
	iss - Increased serum survival
	terC - Tellurium ion resistance protein
	csgA - curlin major subunit CsgA
	fdeC - intimin-like adhesin FdeC
	gad - Glutamate decarboxylase
	hlyE - Avian E. coli haemolysin
	nlpI - lipoprotein NlpI precursor
	(plasmid none)
MLI102	*aaiC* - Type VI secretion protein
	fyuA - Siderophore receptor
	hra - Heat-resistant agglutinin
	irp2 - High molecular weight protein 2 non-ribosomal peptide synthetase
	iutA - Ferric aerobactin receptor
	kpsE - Capsule polysaccharide export inner-membrane protein
	*kpsMII*_K5 - Polysialic acid transport protein; Group 2 capsule
	mchB - Microcin H47 part of colicin H
	mchC - MchC protein
	mchF - ABC transporter protein MchF
	mcmA - Microcin M part of colicin H

pic - serine protease autotransporters of Enterobacteriaceae (SPATE)
sat - Secreted autotransporter toxin
terC - Tellurium ion resistance protein
AslA- Arylsulfatase
capU - Hexosyltransferase homolog
csgA - curlin major subunit CsgA
fdeC - intimin-like adhesin FdeC
fimH - Type 1 fimbriae
gad - Glutamate decarboxylase
hha - hemolysin expression modulator Hha (previous rmoA)
hlyE - Avian E. coli haemolysin
nlpI - lipoprotein NlpI precursor
shiA - homologs of the Shigella flexneri SHI-2 pathogenicity island gene shiA
yehB - Usher, YHD fimbriael cluster
yehC - Chaperone, YHD fimbriael cluster
yehD - Chaperone, Major pilin subunit, YHD fimbriael cluster
(plasmid none)

MLI104-1 *fyuA* - Siderophore receptor
irp2 - High molecular weight protein 2 non-ribosomal peptide synthetase
terC - Tellurium ion resistance protein
AslA- Arylsulfatase
csgA - curlin major subunit CsgA
fimH - Type 1 fimbriae
gad - Glutamate decarboxylase

hlyE - Avian E. coli haemolysin
nlpI - lipoprotein NlpI precursor
yehA - Outer membrane lipoprotein, YHD fimbriael cluster
yehB - Usher, YHD fimbriael cluster
yehC - Chaperone, YHD fimbriael cluster
yehD - Chaperone, Major pilin subunit, YHD fimbriael cluster
(plasmid none)

MLI104-2 *capU* - Hexosyltransferase homolog
fyuA - Siderophore receptor
hra - Heat-resistant agglutinin
irp2 - High molecular weight protein 2 non-ribosomal peptide synthetase
iss - Increased serum survival
iucC - Aerobactin synthetase
iutA - Ferric aerobactin receptor
terC - Tellurium ion resistance protein
AslA - Arylsulfatase
csgA - curlin major subunit CsgA
fdeC - intimin-like adhesin FdeC
fimH - Type 1 fimbriae
gad - Glutamate decarboxylase
hha - hemolysin expression modulator Hha (previous rmoA)
hlyE - Avian E. coli haemolysin
nlpI - lipoprotein NlpI precursor
yehA - Outer membrane lipoprotein, YHD fimbriael cluster

	yehB - Usher, YHD fimbriael cluster
	yehC - Chaperone, YHD fimbriael cluster
	yehD - Chaperone, Major pilin subunit, YHD fimbriael cluster
	(plasmide none)
MLI105	*AslA* - Arylsulfatase
	csgA - curlin major subunit CsgA
	fdeC - intimin-like adhesin FdeC
	fimH - Type 1 fimbriae
	gad - Glutamate decarboxylase
	hha - hemolysin expression modulator Hha (previous rmoA)
	hlyE - Avian E. coli haemolysin
	iss - Increased serum survival
	nlpI - lipoprotein NlpI precursor
	terC - Tellurium ion resistance protein
	yehA - Outer membrane lipoprotein, YHD fimbriael cluster
	yehB - Usher, YHD fimbriael cluster
	yehC - Chaperone, YHD fimbriael cluster
	yehD - Major pilin subunit, YHD fimbriael cluster
	(plasmide none)
MLI106-1	*air* - Enteroaggregative immunoglobulin repeat protein
	chuA - Outer membrane hemin receptor
	eilA - Salmonella HilA homolog

kpsE - Capsule polysaccharide export inner-membrane protein
ompT - Outer membrane protease (protein protease 7)
sitA - Iron transport protein
terC - Tellurium ion resistance protein
AslA - Arylsulfatase
csgA - curlin major subunit CsgA
fdeC - intimin-like adhesin FdeC
fimH - Type 1 fimbriae
gad - Glutamate decarboxylase
hha - hemolysin expression modulator Hha (previous rmoA)
hlyE - Avian E. coli haemolysin
iss - Increased serum survival
nlpI - lipoprotein NlpI precursor
yehA - Outer membrane lipoprotein, YHD fimbriael cluster
yehB - Usher, YHD fimbriael cluster
yehC - Chaperone, YHD fimbriael cluster
yehD - Major pilin subunit, YHD fimbriael cluster
(plasmide none)

MLI106-2 *fyuA* - Siderophore receptor
irp2 - High molecular weight protein 2 non-ribosomal peptide synthetase
iss - Increased serum survival
terC - Tellurium ion resistance protein
AslA - Arylsulfatase
csgA - curlin major subunit CsgA

	fdeC - intimin-like adhesin FdeC
	fimH - Type 1 fimbriae
	gad - Glutamate decarboxylase
	hha - hemolysin expression modulator Hha (previous rmoA)
	hlyE - Avian E. coli haemolysin
	nlpI - lipoprotein NlpI precursor
	yehA - Outer membrane lipoprotein, YHD fimbriael cluster
	yehB - Usher, YHD fimbriael cluster
	yehC - Chaperone, YHD fimbriael cluster
	yehD - Major pilin subunit, YHD fimbriael cluster
p174_MLI106-2	*anr* - AraC negative regulator
	iucC - Aerobactin synthetase
	iutA - Aerobactin synthetase
	sitA - Iron transport protein
	traJ - Protein TraJ (Positive regulator of conjugal transfer operon)
	traT - Outer membrane protein complement resistance
MLI106-3	*air* - Enteroaggregative immunoglobulin repeat protein
	chuA - Outer membrane hemin receptor
	eilA - Salmonella HilA homolog
	gad - Glutamate decarboxylase
	iss - Increased serum survival
	kpsE - Capsule polysaccharide export inner-membrane protein

*kpsMII*_K5 - Polysialic acid transport protein; Group 2 capsule
ompT - Outer membrane protease (protein protease 7)
terC - Tellurium ion resistance protein
AslA - Arylsulfatase
csgA - curlin major subunit CsgA
fdeC - intimin-like adhesin FdeC
fimH - Type 1 fimbriae
hha - hemolysin expression modulator Hha (previous rmoA)
hlyE - Avian E. coli haemolysin
nlpI - lipoprotein NlpI precursor
yehB - Usher, YHD fimbriael cluster
yehC - Chaperone, YHD fimbriael cluster
yehD - Major pilin subunit, YHD fimbriael cluster
(plasmide none)

MLI106-4 *fyuA* - Siderophore receptor
irp2 - High molecular weight protein 2 non-ribosomal peptide synthetase
iss - Increased serum survival
terC - Tellurium ion resistance protein
AslA - Arylsulfatase
csgA - curlin major subunit CsgA
fdeC - intimin-like adhesin FdeC
fimH - Type 1 fimbriae
gad - Glutamate decarboxylase
hha - hemolysin expression modulator Hha (previous rmoA)

p174_MLI106-4	*hlyE* - Avian E. coli haemolysin
	nlpI - lipoprotein NlpI precursor
	yehA - Outer membrane lipoprotein, YHD fimbriael cluster
	yehB - Usher, YHD fimbriael cluster
	yehC - Chaperone, YHD fimbriael cluster
	yehD - Major pilin subunit, YHD fimbriael cluster
	anr - AraC negative regulator
	iucC - Aerobactin synthetase
	iutA - Aerobactin synthetase
	sitA - Iron transport protein
	traJ - Protein TraJ (Positive regulator of conjugal transfer operon)
	traT - Outer membrane protein complement resistance
MLI107	*gad* - Glutamate decarboxylase
	iss - Increased serum survival
	lpfA - Long polar fimbriae
	terC - Tellurium ion resistance protein
	csgA - curlin major subunit CsgA
	fdeC - intimin-like adhesin FdeC
	fimH - Type 1 fimbriae
	hlyE - Avian E. coli haemolysin
	nlpI - lipoprotein NlpI precursor
	yehA - Outer membrane lipoprotein, YHD fimbriael cluster

	yehB - Usher, YHD fimbriael cluster
	yehC - Chaperone, YHD fimbriael cluster
	yehD - Major pilin subunit, YHD fimbriael cluster
	(plasmid none)
MLI108-1	*terC* - Tellurium ion resistance protein
	AslA - Arylsulfatase
	csgA - curlin major subunit CsgA
	fdeC - intimin-like adhesin FdeC
	fimH - Type 1 fimbriae
	gad - Glutamate decarboxylase
	hha - hemolysin expression modulator Hha (previous rmoA)
	hlyE - Avian E. coli haemolysin
	nlpI - lipoprotein NlpI precursor
	yehA - Outer membrane lipoprotein, YHD fimbriael cluster
	yehB - Usher, YHD fimbriael cluster
	yehC - Chaperone, YHD fimbriael cluster
	yehD - Major pilin subunit, YHD fimbriael cluster
p172_MLI108-1	*anr* - AraC negative regulator
	iucC - Aerobactin synthetase
	iutA - Aerobactin synthetase
	sitA - Iron transport protein
	traJ - Protein TraJ (Positive regulator of conjugal transfer operon)
	traT - Outer membrane protein complement resistance

MLI108-2	*terC* - Tellurium ion resistance protein
	AslA - Arylsulfatase
	csgA - curlin major subunit CsgA
	fdeC - intimin-like adhesin FdeC
	fimH - Type 1 fimbriae
	gad - Glutamate decarboxylase
	hha - hemolysin expression modulator Hha (previous rmoA)
	hlyE - Avian E. coli haemolysin
	nlpI - lipoprotein NlpI precursor
	yehA - Outer membrane lipoprotein, YHD fimbriael cluster
	yehB - Usher, YHD fimbriael cluster
	yehC - Chaperone, YHD fimbriael cluster
	yehD - Major pilin subunit, YHD fimbriael cluster
	anr - AraC negative regulator
p172_MLI108-2	*iucC* - Aerobactin synthetase
	iutA - Aerobactin synthetase
	sitA - Iron transport protein
	traJ - Protein TraJ (Positive regulator of conjugal transfer operon)
	traT - Outer membrane protein complement resistance
MLI108-3	*air* - Enteroaggregative immunoglobulin repeat protein
	chuA - Outer membrane hemin receptor
	eilA - Salmonella HilA homolog

	gad - Glutamate decarboxylase
	iss - Increased serum survival
	kpsE - Capsule polysaccharide export inner-membrane protein
	*kpsMII*_K5 - Polysialic acid transport protein; Group 2 capsule
	ompT - Outer membrane protease (protein protease 7)
	sitA - Iron transport protein
	terC - Tellurium ion resistance protein
	AslA - Arylsulfatase
	csgA - curlin major subunit CsgA
	fdeC - intimin-like adhesin FdeC
	fimH - Type 1 fimbriae
	hha - hemolysin expression modulator Hha (previous rmoA)
	hlyE - Avian E. coli haemolysin
	nlpI - lipoprotein NlpI precursor
	yehA - Outer membrane lipoprotein, YHD fimbriael cluster
	yehB - Usher, YHD fimbriael cluster
	yehC - Chaperone, YHD fimbriael cluster
	yehD - Major pilin subunit, YHD fimbriael cluster
	anr - AraC negative regulator
p89_MLI108-3	*capU* - Hexosyltransferase homolog
	traJ - Protein TraJ (Positive regulator of conjugal transfer operon)
	traT - Outer membrane protein complement resistance
MLI109	*air* - Enteroaggregative immunoglobulin repeat protein
	chuA - Outer membrane hemin receptor

	eilA - Salmonella HilA homolog
	fyuA - Siderophore receptor
	gad - Glutamate decarboxylase
	hra - Heat-resistant agglutinin
	irp2 - High molecular weight protein 2 non-ribosomal peptide synthetase
	iss - Increased serum survival
	kpsE - Capsule polysaccharide export inner-membrane protein
	*kpsMII*_K5 - Polysialic acid transport protein; Group 2 capsule
	terC - Tellurium ion resistance protein
	AslA - Arylsulfatase
	csgA - curlin major subunit CsgA
	fdeC - intimin-like adhesin FdeC
	fimH - Type 1 fimbriae
	hha - hemolysin expression modulator Hha (previous rmoA)
	hlyE - Avian E. coli haemolysin
	nlpI - lipoprotein NlpI precursor
	yehB - Usher, YHD fimbriael cluster
	yehC - Chaperone, YHD fimbriael cluster
	yehD - Major pilin subunit, YHD fimbriael cluster
	ORF3 - Isoprenoid Biosynthesis
p140_MLI109	*ORF*4 - Putative isopentenyl-diphosphate delta-isomerase
	aap - Dispersin, antiaggregation protein
	aar - AggR-activated regulator
	aatA - Dispersin transporter protein
	afaD - Afimbrial adhesion

agg3C - Usher, AAF/III assembly unit
agg3D - Chaperone, AAF/III assembly unit
agg5A - Aggregative adherence fimbria V major subunit Agg5A
aggR - AraC transcriptional activator
anr - AraC negative regulator
traT - Outer membrane protein complement resistance

MLI110	*iss* - Increased serum survival
	terC - Tellurium ion resistance protein
	AslA - Arylsulfatase
	fdeC - intimin-like adhesin FdeC
	gad - Glutamate decarboxylase
	hlyE - Avian E. coli haemolysin
	nlpI - lipoprotein NlpI precursor
	yehA - Outer membrane lipoprotein, YHD fimbriael cluster
	yehB - Usher, YHD fimbriael cluster
	yehC - Chaperone, YHD fimbriael cluster
	yehD - Major pilin subunit, YHD fimbriael cluster
	shiA - homologs of the Shigella flexneri SHI-2 pathogenicity island gene shiA
	(plasmid none)

MLI114	*aaiC* - Type VI secretion protein
	afaD - Afimbrial adhesion
	air - Enteroaggregative immunoglobulin repeat protein
	chuA - Outer membrane hemin receptor
	eilA - Salmonella HilA homolog
	fyuA - Siderophore receptor
	gad - Glutamate decarboxylase
	irp2 - High molecular weight protein 2 non-ribosomal peptide synthetase
	kpsE - Capsule polysaccharide export inner-membrane protein
	kpsMII - Polysialic acid transport protein; Group 2 capsule
	pic - serine protease autotransporters of Enterobacteriaceae (SPATE)
	terC - Tellurium ion resistance protein
	AslA - Arylsulfatase
	csgA - curlin major subunit CsgA
	fdeC - intimin-like adhesin FdeC
	fimH - Type 1 fimbriae
	hha - hemolysin expression modulator Hha (previous rmoA)
	hlyE - Avian E. coli haemolysin
	nlpI - lipoprotein NlpI precursor
	yehA - Outer membrane lipoprotein, YHD fimbriael cluster
	yehB - Usher, YHD fimbriael cluster
	yehC - Chaperone, YHD fimbriael cluster
	yehD - Major pilin subunit, YHD fimbriael cluster
	(plasmid none)

MLI121	*AslA* - Arylsulfatase<br>*aaiC* - Type VI secretion protein<br>*afaD* - Afimbrial adhesion<br>*air* - Enteroaggregative immunoglobulin repeat protein<br>*chuA* - Outer membrane hemin receptor<br>*csgA* - curlin major subunit CsgA<br>*eilA* - Salmonella HilA homolog<br>*fdeC* - intimin-like adhesin FdeC<br>*fimH* - Type 1 fimbriae<br>*fyuA* - Siderophore receptor<br>*gad* - Glutamate decarboxylase<br>*hha* - hemolysin expression modulator Hha (previous rmoA)<br>*hlyE* - Avian E. coli haemolysin<br>*irp2* - High molecular weight protein 2 non-ribosomal peptide synthetase<br>*kpsE* - Capsule polysaccharide export inner-membrane protein<br>*kpsMII* - Polysialic acid transport protein; Group 2 capsule<br>*nlpI* - lipoprotein NlpI precursor<br>*pic* - serine protease autotransporters of Enterobacteriaceae (SPATE)<br>*terC* - Tellurium ion resistance protein<br>*yehA* - Outer membrane lipoprotein, YHD fimbriael cluster<br>*yehB* - Usher, YHD fimbriael cluster<br>*yehC* - Chaperone, YHD fimbriael cluster<br>*yehD* - Major pilin subunit, YHD fimbriael cluster<br>(plasmide none)

MLI124	*capU* - Hexosyltransferase homolog
	gad - Glutamate decarboxylase
	iss - Increased serum survival
	lpfA - Long polar fimbriae
	terC - Tellurium ion resistance protein
	csgA - curlin major subunit CsgA
	fdeC - intimin-like adhesin FdeC
	hha - hemolysin expression modulator Hha (previous rmoA)
	hlyE - Avian E. coli haemolysin
	nlpI - lipoprotein NlpI precursor
	yehA - Outer membrane lipoprotein, YHD fimbriael cluster
	yehB - Usher, YHD fimbriael cluster
	yehC - Chaperone, YHD fimbriael cluster
	yehD - Major pilin subunit, YHD fimbriael cluster
p68_MLI124	*cia* - Colicin ia
	traT - Outer membrane protein complement resistance
	astA - Heat-stable enterotoxin EAST-1
p15_MLI124	*eatA* - Mucin-degrading serine protease autotransporters of Enterobacteriaceae and ETEC (SPATE)

Supplementary table 3: Accession/SRA, biosample ID, bioproject IDs, and geographic sample origin of the *E. coli* isolates included in the additional cgMLST analysis to estimate the likelihood of importation from Europe for the *E. coli* strains isolated in Mali.

***E. coli* Isolate**	**accession/SRA**	**biosample**	**bioproject(s)**	**origin**
BNI_Iso00299	SRS8179310	SAMN17766615	PRJNA699140	Ghana
BNI_Iso00270	SRS8179297	SAMN17766603	PRJNA699140<br>PRJNA822931	Ghana
BNI_Iso00245	SRS8179293	SAMN17766599	PRJNA699140	Ghana
BNI_Iso00225	SRS8179283	SAMN17766590	PRJNA699140<br>PRJNA822931	Ghana
BNI_Iso00222	SRS8179282	SAMN17766589	PRJNA699140	Ghana
BNI_Iso00221	SRS8179281	SAMN17766588	PRJNA699140	Ghana
BNI_Iso00176	SRS8179261	SAMN17766570	PRJNA699140	Ghana
BNI_Iso00152	SRS8179254	SAMN17766564	PRJNA699140	Ghana
BNI_Iso00117	SRS8179246	SAMN17766557	PRJNA699140	Ghana
BNI_Iso00068	SRS8179303	SAMN17766546	PRJNA699140	Ghana

BNI_Iso00049	SRS8179280	SAMN17766544	PRJNA699140	Ghana
BNI_Iso00041	SRS8179259	SAMN17766542	PRJNA699140<br>PRJNA822931	Ghana
9_01-2	ERS10153379	SAMEA12546824	PRJEB50347<br>PRJNA514245	Niger
9_01-1	ERS10153378	SAMEA12546823	PRJEB50347<br>PRJNA514245	Niger
8-05	ERS10153377	SAMEA12546822	PRJEB50347<br>PRJNA514245	Niger
8_14	ERS10153376	SAMEA12546821	PRJEB50347<br>PRJNA514245	Niger
8_11	ERS10153375	SAMEA12546820	PRJEB50347<br>PRJNA514245	Niger
8_10	ERS10153374	SAMEA12546819	PRJEB50347<br>PRJNA514245	Niger
7_24	ERS10153373	SAMEA12546818	PRJEB50347	Niger

			PRJNA514245	
7_17	ERS10153372	SAMEA12546817	PRJEB50347<br>PRJNA514245	Niger
7_05-2	ERS10153371	SAMEA12546816	PRJEB50347<br>PRJNA514245	Niger
7_05-1	ERS10153370	SAMEA12546815	PRJEB50347<br>PRJNA514245	Niger
9_12	ERS10153381	SAMEA12546826	PRJEB50347<br>PRJNA514245	Niger
9_02	ERS10153380	SAMEA12546825	PRJEB50347<br>PRJNA514245	Niger
C725_88	SRS14726881	SAMN02436011	PRJNA871208	Germany
17-06461	SRS9240773	SAMN19776852	PRJNA739205<br>PRJNA514245	Germany
17-06334	SRS9240772	SAMN19776851	PRJNA739205<br>PRJNA514245	Germany

17-05751	SRS9240771	SAMN19776850	PRJNA739205<br>PRJNA514245	Germany
17-05723	SRS9240770	SAMN19776849	PRJNA739205<br>PRJNA514245	Germany
17-05720	SRS9240769	SAMN19776848	PRJNA739205<br>PRJNA514245	Germany
17-04777	SRS9240767	SAMN19776847	PRJNA739205<br>PRJNA514245	Germany
17-04317	SRS9240768	SAMN19776846	PRJNA739205<br>PRJNA514245	Germany
17-04189	SRS9240766	SAMN19776845	PRJNA739205<br>PRJNA514245	Germany
17-04145	SRS9240764	SAMN19776844	PRJNA739205<br>PRJNA514245	Germany
17-04144	SRS9240763	SAMN19776843	PRJNA739205<br>PRJNA514245	Germany

17-01866	SRS9240762	SAMN19776842	PRJNA739205<br>PRJNA514245	Germany
17-01717	SRS9240761	SAMN19776841	PRJNA739205<br>PRJNA514245	Germany
17-01338	SRS9240757	SAMN19776838	PRJNA739205<br>PRJNA514245	Germany
17-01275	SRS9240758	SAMN19776837	PRJNA739205<br>PRJNA514245	Germany
17-01274	SRS9240756	SAMN19776836	PRJNA739205<br>PRJNA514245	Germany
04-01674	ERS3125481	SAMEA5318244	PRJEB31106<br>PRJNA514245	Germany
03-3095	ERS3125480	SAMEA5318243	PRJEB31106<br>PRJNA514245	Germany
03-08411	ERS3125479	SAMEA5318242	PRJEB31106<br>PRJNA514245	Germany

03-00945	ERS3125478	SAMEA5318241	PRJEB31106<br>PRJNA514245	Germany
00-03365	ERS3125477	SAMEA5318240	PRJEB31106<br>PRJNA514245	Germany
05-06739	ERS3125484	SAMEA5318247	PRJEB31106<br>PRJNA514245	Germany
05-00826	ERS3125483	SAMEA5318246	PRJEB31106<br>PRJNA514245	Germany
04-03909	ERS3125482	SAMEA5318245	PRJEB31106<br>PRJNA514245	Germany

Supplementary Figure 1: Ridom SeqSphere+ generated cgMLST-based minimum spanning tree showing the genetic distance of the *E. coli* isolates indicated by numbers of allele differences in a total of 2274 assessed columns (distance based on STEC cgMLST: 2274; MST cluster distance threshold 10). The nodes are colored by country of origin: red = Germany, green = Ghana, violet = Niger, and light blue = Mali.

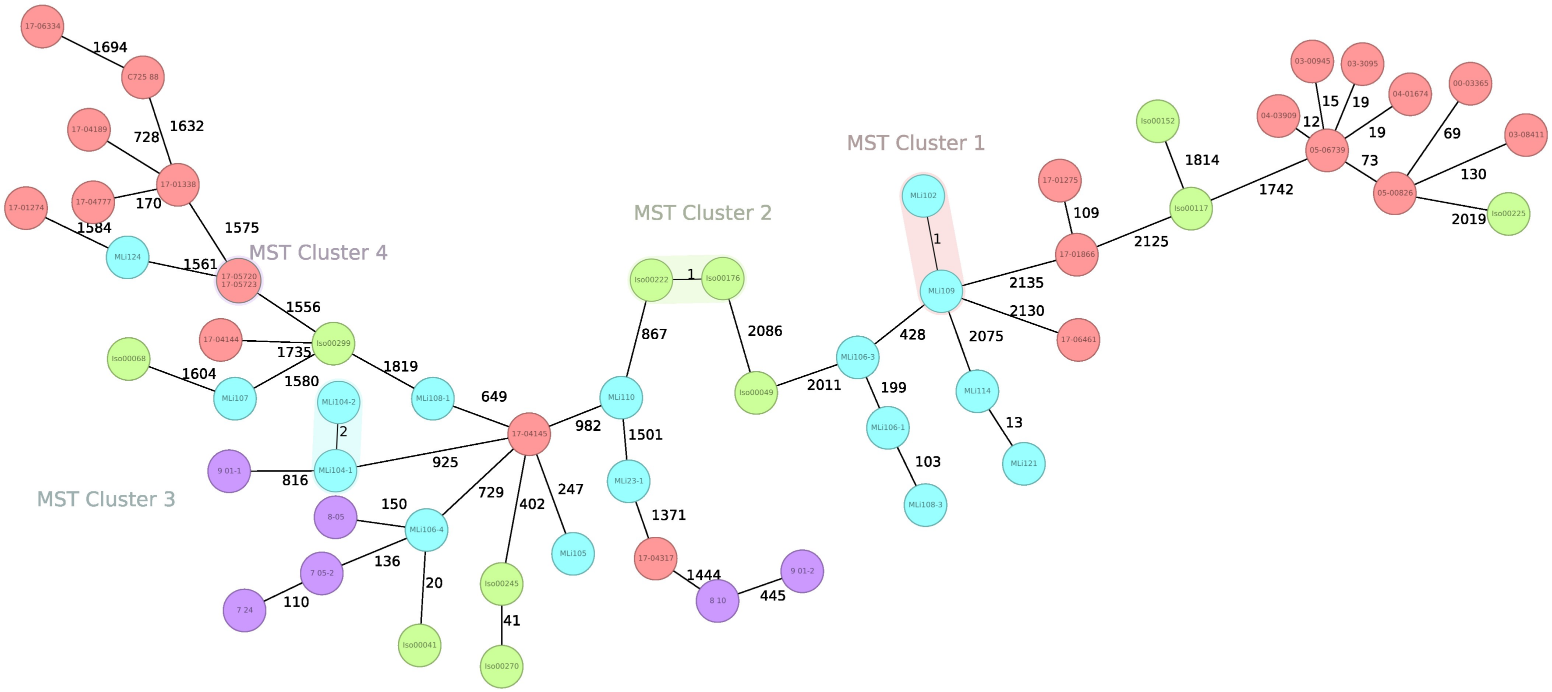

Supplementary Figure 2: Heat-map of the associations of bacterial isolates with chromosomal as well as plasmid-encoded ARGs. A light green rectangle indicates the presence of a specific ARG on a bacterial plasmid. A red rectangle indicates the presence of a specific ARG on the bacterial chromosome of the *E. coli* isolate given in the header of the heat map. A grey rectangle indicates the absence of a particular ARG.

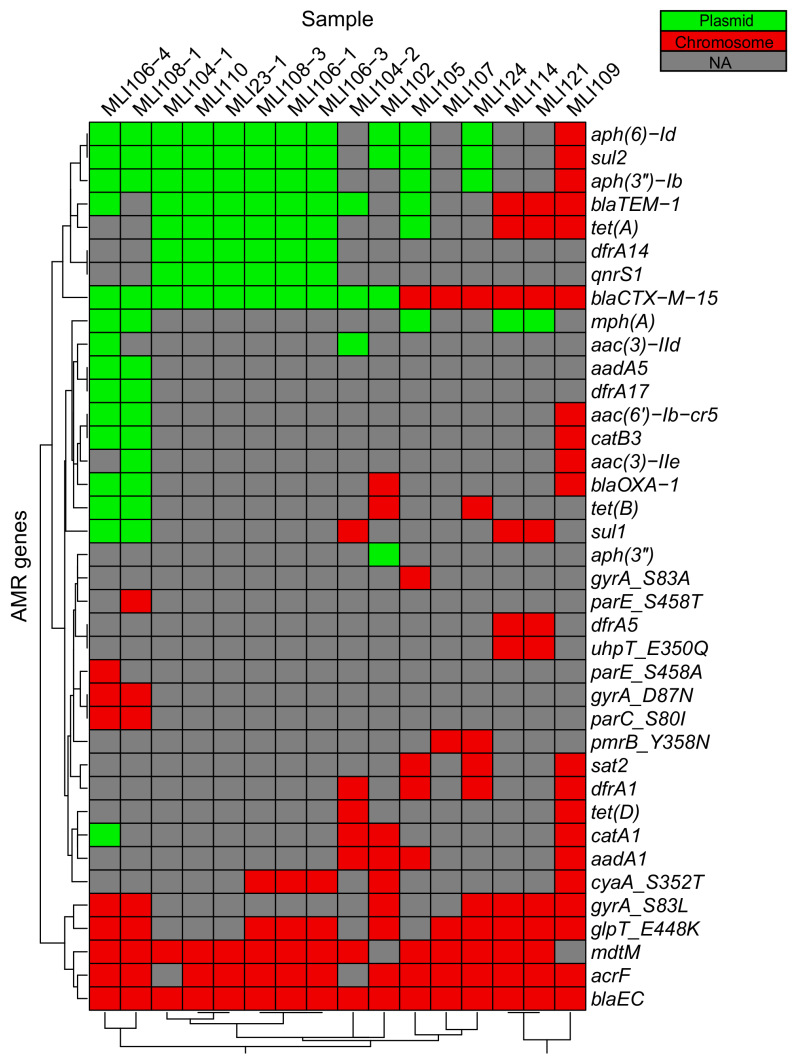

Supplement: Supplementary file 1 [file Table_1.pdf]
