# Supplementary figures and images for: Molecular epidemiology of enterically colonizing Escherichia coli with resistance against third-generation cephalosporins isolated from stool samples of European soldiers with concomitant diarrhea on deployment in Western African Mali
Source: Front Microbiol. 2023 May 5;14:1169829. doi: 10.3389/fmicb.2023.1169829 (PMC10198576; doi:10.3389/fmicb.2023.1169829)

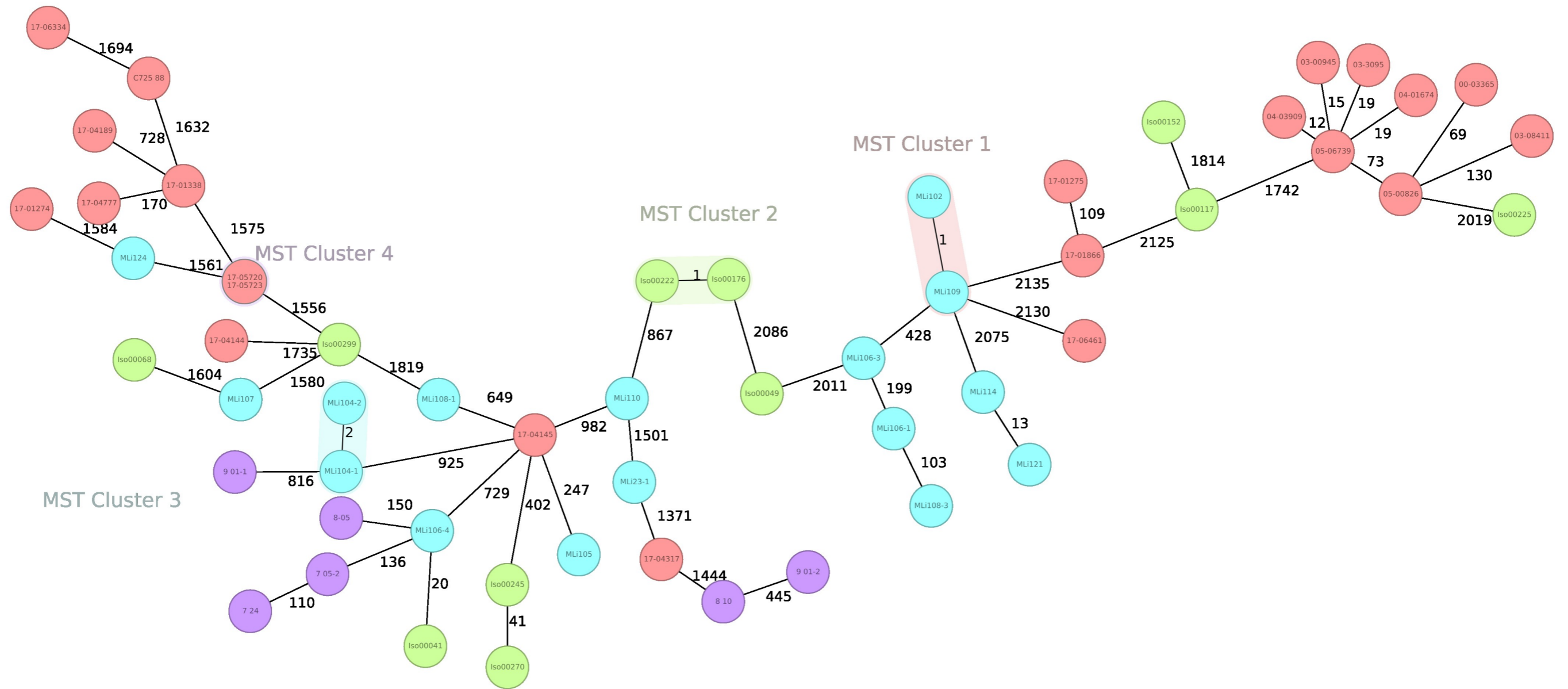

Supplement: Supplementary file 3 [file Image_1.PDF]

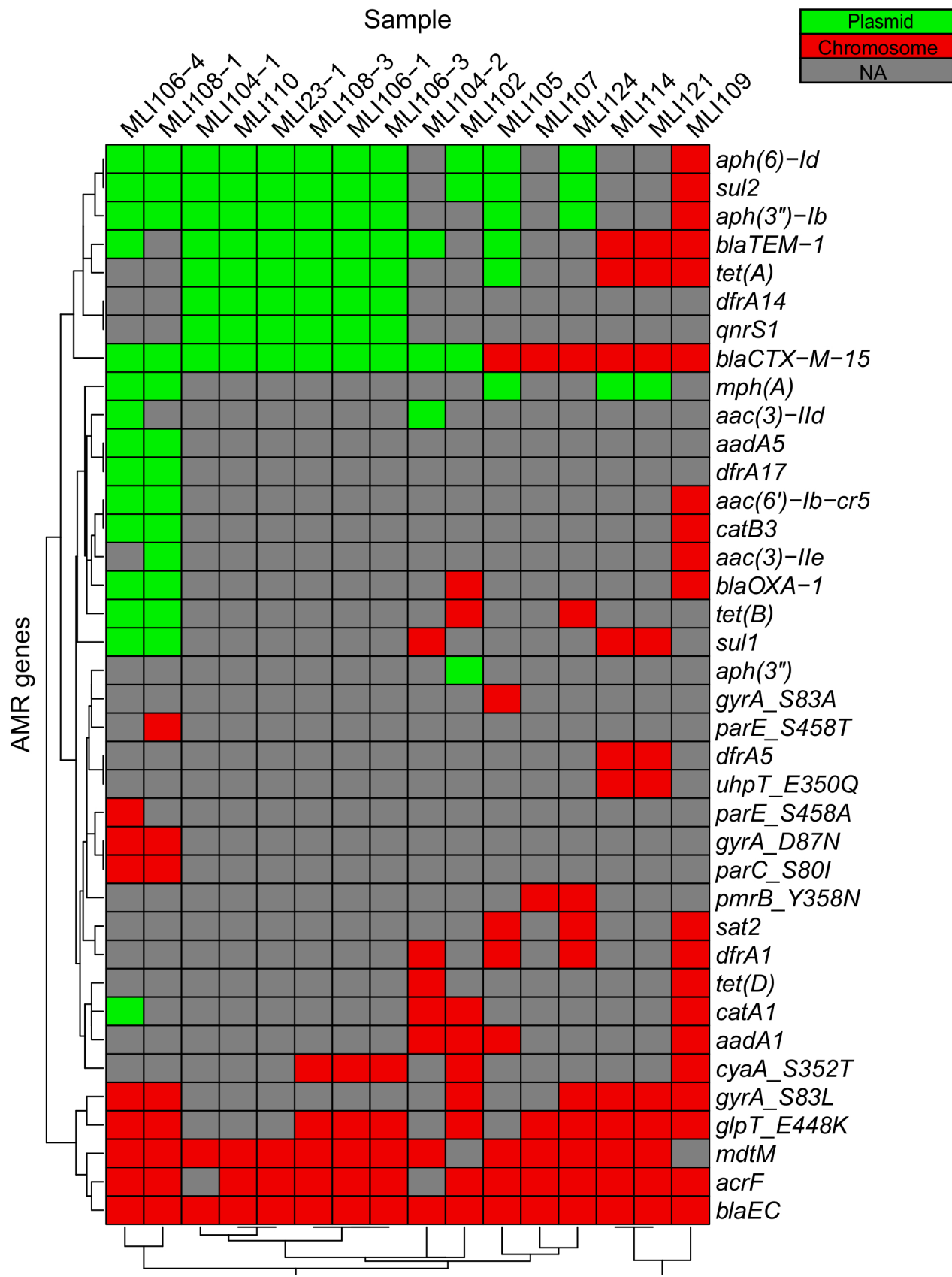

Supplement: Supplementary file 4 [file Image_2.PDF]
